# Structure of *Vibrio* collagenase VhaC provides insight into the mechanism of bacterial collagenolysis

**DOI:** 10.1038/s41467-022-28264-1

**Published:** 2022-01-28

**Authors:** Yan Wang, Peng Wang, Hai-Yan Cao, Hai-Tao Ding, Hai-Nan Su, Shi-Cheng Liu, Guangfeng Liu, Xia Zhang, Chun-Yang Li, Ming Peng, Fuchuan Li, Shengying Li, Yin Chen, Xiu-Lan Chen, Yu-Zhong Zhang

**Affiliations:** 1 State Key Laboratory of Microbial Technology, Shandong University, Qingdao, 266237 China; 2 College of Marine Life Sciences, and Frontiers Science Center for Deep Ocean Multispheres and Earth System, Ocean University of China, Qingdao, 266003 China; 3 Laboratory for Marine Biology and Biotechnology, Pilot National Laboratory for Marine Science and Technology, Qingdao, 266237 China; 4 Antarctic Great Wall Ecology National Observation and Research Station, Polar Research Institute of China, Shanghai, 200136 China; 5 Department of Molecular Biology, Qingdao Vland Biotech Inc., Qingdao, China; 6 National Center for Protein Science Shanghai, Shanghai Advanced Research Institute, Chinese Academy of Sciences, Shanghai, 201210 China; 7 National Glycoengineering Research Center and Shandong Key Laboratory of Carbohydrate Chemistry and Glycobiology, Shandong University, Qingdao, China; 8 School of Life Sciences, University of Warwick, Coventry, UK; 9 Marine Biotechnology Research Center, State Key Laboratory of Microbial Technology, Shandong University, Qingdao, 266237 China

**Keywords:** Proteolysis, Enzyme mechanisms, Proteases, X-ray crystallography, SAXS

## Abstract

The collagenases of *Vibrio* species, many of which are pathogens, have been regarded as an important virulence factor. However, there is little information on the structure and collagenolytic mechanism of *Vibrio* collagenase. Here, we report the crystal structure of the collagenase module (CM) of *Vibrio* collagenase VhaC and the conformation of VhaC in solution. Structural and biochemical analyses and molecular dynamics studies reveal that triple-helical collagen is initially recognized by the activator domain, followed by subsequent cleavage by the peptidase domain along with the closing movement of CM. This is different from the peptidolytic mode or the proposed collagenolysis of *Clostridium* collagenase. We propose a model for the integrated collagenolytic mechanism of VhaC, integrating the functions of VhaC accessory domains and its collagen degradation pattern. This study provides insight into the mechanism of bacterial collagenolysis and helps in structure-based drug design targeting of the *Vibrio* collagenase.

## Abstract (web summary)

The collagenolytic mechanism of *Vibrio* collagenase, a virulence factor, remains unclear. Here, the authors report the structure of *Vibrio* collagenase VhaC and propose the mechanism for collagen recognition and degradation, providing new insight into bacterial collagenolysis.

## Introduction

Collagens are the most abundant proteins in mammals, mainly in the extracellular matrix (ECM)^1^. Of the 28 types of collagens, type I collagen is the most widely occurring and has a hierarchical structure^2^. Monomeric type I collagen (tropocollagen, ~300 nm in length and ~1.5 nm in diameter) is a righthanded triple helix comprising three α chains (two α1 chains and one α2 chain). Each chain consists of Gly-X-Y repeating triplets where X and Y are often occupied by proline and hydroxyproline, respectively. The N- and C-terminal telopeptides, located on the flanks of the triple-helical region, constitute the non-triple helical regions of tropocollagen^1^. Every five tropocollagens form one quasi-hexagonal microfibril unit^3,4^. Microfibrils are interdigitated regularly with each other, forming mature collagen fibrils. Various covalent cross-links, such as pyridinium compounds and pyrroles within or between microfibrils, maintain the strength and stability of the fibrils^5^. Collagen fibrils further aggregate with proteoglycans to form collagen fibers and other super structures in the ECM^3,6,7^. Due to its tight structure and hierarchical assembly, collagen can only be degraded by a very few proteases^8^.

Collagenolytic proteases, capable of hydrolyzing native collagen under physiological conditions, are found in animals and microbes. Mammalian matrix metalloproteinases (MMPs), belonging to the M10A subfamily, play crucial roles in the interstitial collagen catabolism and their structures and collagenolytic mechanisms have been extensively studied^9–11^, especially for MMP-1. MMP-1 cleaves type I tropocollagen chain at a single Gly-Ile/Leu site generating 1/4 and 3/4 length fragments via a complicated molecular mechanism^12–18^. Bacterial collagenolytic proteases, that are capable of hydrolyzing native collagen at multiple sites, are distributed in the M9, S1, and S8 MEROPS peptidase families, some of which are strongly linked to bacterial pathogenesis^19–21^. Most known bacterial collagenases belong to the M9 family, which is subdivided into the M9A (*Vibrio* collagenases) and M9B (*Clostridium* collagenases) subfamilies. Up to now, information on the three-dimensional structure of *Vibrio* collagenase is still lacking. As for *Clostridium* collagenases, only the crystal structure of the collagenase module (CM) of ColG^22^ and those of the peptidase domains of ColH and ColT have been solved^23^. Eckhard et al.^22^ proposed a “chew and digest” collagenolytic model based on the saddle-shaped architecture of the CM of ColG. In this model, triple-helical collagen initially contacts the peptidase domain in the open state; in the closed state, triple-helical collagen interacts with both the activator and the peptidase domains in the CM and then is unwound and degraded progressively. However, due to the lack of sufficient experimental evidence for this model, how triple-helical collagen is recognized by the activator and the peptidase domains is still largely unclear. Thus, the mechanism of collagen recognition by M9 bacterial collagenases warrants further study in order to better understand its role in pathogenesis.

Collagenases of certain pathogenic *Vibrio* species accelerate bacterial dissemination and facilitate diffusion of other toxins through hydrolysis of the collagenous components of the ECM, they are therefore regarded as an important virulence factor^24^. So far, studies have largely been focused on the physicochemical properties^25–28^, cytotoxicity, and fish pathogenicity^29^ of *Vibrio* collagenases. Except for an early report that *Vibrio* collagenase attacked the collagen molecule at 3/4 from the N-terminus by digesting the preferential peptide bond, Y-Gly, in the first step of collagen degradation^30^, information on the collagen cleavage pattern of *Vibrio* collagenase is scarce. Like *Clostridium* collagenases, the characteristic domain architecture of *Vibrio* collagenase consists of a peptidase M9N domain (activator domain) and a peptidase M9 domain (peptidase domain), forming a CM^26,31^. Some *Vibrio* collagenases contain additional C-terminal accessory domains, such as the polycystic kidney disease-like domain (PKD-like domain) and the bacterial prepeptidase C-terminal domain (PPC domain)^8,20^. The PPC domain of *Vibrio* collagenase Ghcol from *Grimontia hollisae* (formerly known as *Vibrio hollisae*) has been demonstrated to function as a collagen binding domain (CBD)^32^. Despite these studies, little is known about the mechanism of *Vibrio* collagenase for collagen recognition and degradation due to the lack of structural information.

In this work, we report the structure and propose the collagenolytic mechanism of a *Vibrio* collagenase VhaC from *Vibrio harveyi* VHJR7, which is isolated from an infected fish^33,34^. Protease VhaC is a multi-domain enzyme composed of a CM containing an activator domain and a peptidase domain, a PKD-like domain and a PPC domain. The crystal structure of the CM is solved at 1.8 Å resolution and the overall conformation and interdomain arrangement of VhaC in solution is determined by small angle x-ray scattering (SAXS). The mechanism of VhaC for the recognition and catalysis of triple-helical collagen is illustrated based on structural and biochemical analyses and molecular dynamics studies, and the functions of the C-terminal accessory PKD-like and PPC domains in collagenolysis are analyzed. Finally, combined with its collagen degradation pattern, a model for collagen fibers degradation by VhaC is proposed. These results reveal the structure of *Vibrio* collagenase and its mechanism for collagen recognition and degradation, providing insight into the mechanism of bacterial collagenolysis and helping in the development of strategies to prevent and treat infection caused by pathogenic *Vibrio* species.

## Results

### Characterization and structural analysis of VhaC

The *vhaC* gene from *Vibrio harveyi* VHJR7 was predicted to encode a putative M9A collagenase (GenBank accession No. WP_047516938.1) of 814 amino acid residues. Mature VhaC protein contains 745 residues from Ser70 to Gln814 with the N-terminal signal peptide and propeptide being removed, which presented as a monomer in solution (Supplementary Fig. 1a). VhaC had activity toward various types of collagens (Supplementary Table 1), showing that it is a collagenase. With type I collagen as the substrate, VhaC has optimal enzymatic reaction conditions at 40 °C and pH 8.0 (Supplementary Fig. 1b, c).

**Fig. 1:**
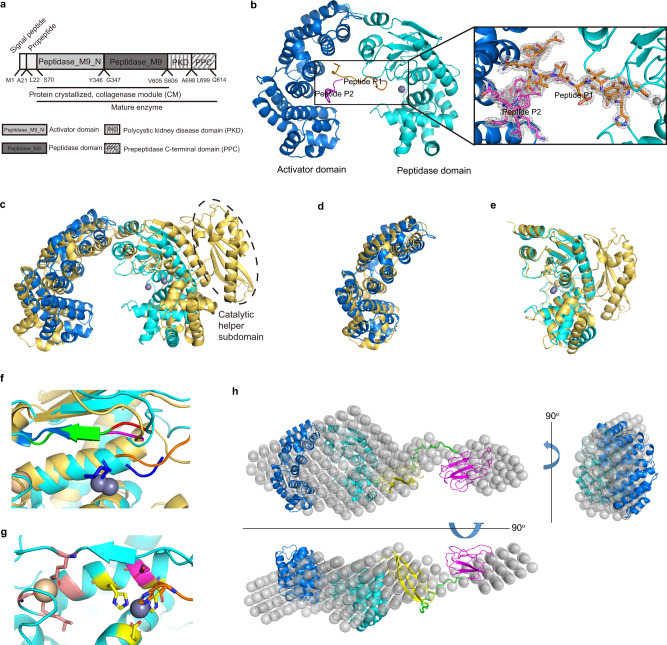
Crystal structure of the CM and overall conformation of VhaC in solution. **a** Schematic diagram of the domain organization of VhaC precursor. **b** The overall structure of CM. The activator domain is shown in marine, and the peptidase domain in cyan. Zn^2+^ (gray) and Ca^2+^ (wheat) are shown as spheres. Peptides P1 and P2 derived from the propeptide are colored in orange and magenta, respectively. A close-up view of the 2*F*_o_-*F*_c_ omit electron density map of the peptide P1 (orange sticks), peptide P2 (magenta sticks) and Zn^2+^ ion (gray sphere) contoured at 1.0 *σ* (gray) is shown. **c** A comparison of the CM structures between VhaC and ColG (golden) (PDB: 2Y50). The catalytic helper subdomain of ColG is indicated by dotted ellipse. **d** Structural superimposition of the activator domains of VhaC (marine) and ColG (golden). **e** Structural superimposition of the peptidase domains of VhaC (cyan) and ColG (golden). **f** Structural superposition of the catalytic centers of CM and 2Y6I. Peptide P1 in CM is colored in orange and isoamyl-phosphonyl-Gly-Pro-Ala in 2Y6I is colored in blue. The double-Gly motif representing the S1′ recognition site is shown in red (CM) and magenta (2Y6I). The edge strand representing the non-primed substrate-recognition site is shown in green (CM) and marine (2Y6I). **g** Ribbon representation of the catalytic center of CM. Residues His477, His481, Glu505 (shown in yellow), and histidine from peptide P1 (shown in orange) tetrahedrally coordinate the catalytic Zn^2+^ (gray sphere). The catalytic Glu478 is shown in magenta. The Ca^2+^ is coordinated by Gly485, Leu489, Gly491, and Glu446 (shown in salmon). **h** Superimposition between the ab initio beads model and the rigid body model of VhaC. The beads model is represented as grey spheres. The rigid body model is drawn as a ribbon representation and colored marine (the activator domain), cyan (the peptidase domain), yellow (the PKD-like domain), and magenta (the PPC domain). The poly-glycine linker added by CORAL is shown in green.

Sequence analysis using Conserved Domain Search showed that VhaC is a multi-domain protein containing an activator domain (Ser70-Tyr346), a peptidase domain (Gly347-Val605), a PKD-like domain (Ser606-Ala698), and a PPC domain (Leu699-Gln814) (Fig. 1a). The activator domain and the peptidase domain form the CM. To obtain the structure of VhaC, the full-length VhaC (Met1-Gln814) and its CM (Met1-Val605) were expressed, and an attempt was made to crystallize the recombinant proteins. However, only the crystal structure of CM was solved to 1.8 Å resolution. Data collection statistics are shown in Supplementary Table 2. The CM crystal belongs to the *P* 2_1_ space group with one molecule per asymmetric unit. The CM structure contains residues from Cys78 to His609, suggesting that the N-terminal signal peptide and propeptide in the CM precursor are cleaved off during enzyme maturation. The eight N-terminal residues from Ser70 to Val77 were not observed in the CM structure due to the ambiguous electron density. The CM structure presents a saddle-shaped architecture (Fig. 1b), which is similar to that of ColG (PDB: 2Y50) but with a more contracted catalytic cavity (Fig. 1c). The activator domain of VhaC is similar to that of ColG with a root mean square deviation (RMSD) of 3.861 Å (Fig. 1d). The peptidase domain of VhaC is also similar to that of ColG with an RMSD value of 1.720 Å (Fig. 1e). The CM of ColG contains an additional catalytic helper subdomain (Fig. 1c), which is a part of the peptidase domain and indispensable for the full enzymatic activity of ColG^22,35^. However, this domain is absent from VhaC, suggesting that differences may exist in the collagen degradation mechanisms of the M9A and M9B subfamilies.

Unexpectedly, the structure data show that the CM molecule binds two peptides (peptide P1: 24-EPSQQVTEIYQHHA-37; peptide P2: 48-DYAPTKLLPQQP-59) derived from the propeptide of VhaC via hydrogen bonds and hydrophobic interactions (Fig. 1b). While peptide P2 entirely binds to the activator domain, the N-terminus of peptide P1 binds to the activator domain and its C-terminus extends and binds to the catalytic center of the peptidase domain (Fig. 1b). As shown in Fig. 1f, the complex structure of the CM of ColG with isoamyl-phosphonyl-Gly-Pro-Ala (PDB: 2Y6I) defines the S1′ peptide recognition site formed by a double-Gly motif (Gly493-Gly494)^22^. Correspondingly, in the CM structure of VhaC, the C-terminal residues of peptide P1 also bind to the conserved Gly441-Gly442 motif, representing the S1′ peptide recognition site (Fig. 1f). Therefore, peptides P1 and P2 can be regarded as substrate analogues in the CM structure, which may provide clues for the recognition pattern of collagen substrates in CM. In addition, as in ColG (495-LYIE-498), an edge strand (443-MYIE-446) of VhaC forms the canonical non-primed substrate-recognition site with the double-Gly motif as the entry (Fig. 1f). In the CM structure, a catalytic Zn^2+^ is tetrahedrally coordinated by the side chains of His477, His481, and Glu505 from the peptidase domain, as well as the side chain of a histidine from peptide P1 (Fig. 1g) instead of the characteristic water molecule observed in 2Y50^22^. In addition, a Ca^2+^ ion, located on the left rim of the active-site cleft and 14.2 Å away from the catalytic Zn^2+^ ion, is coordinated by the backbone oxygens of Gly485, Leu489, and Gly491 and the side chain of Glu446 (Fig. 1g). The Ca^2+^ ion is known to stabilize the Zn^2+^ ion, and as such it is essential for the enzymatic activity of M9B collagenase^23,36^.

Because the structure of full-length VhaC could not be obtained via crystallization, VhaC was alternatively subjected to SAXS measurement to analyze its overall conformation and interdomain arrangement in solution. Related SAXS-derived molecular parameters are shown in Supplementary Table 3 and Supplementary Fig. 2a, b. The ab initio beads model shows that the overall shape of VhaC is long and flat, with a wide head and a short tail; dimensions are estimated to be ~176 Å × 63 Å × 28 Å (Fig. 1h). To estimate the interdomain arrangement of VhaC, a rigid body model was built using the crystal structure of CM and homology models of PKD-like and PPC domains. The resulting rigid body model fits well to the ab initio beads model and suggests a side-by-side assembly of each domain (Fig. 1h). The theoretical scattering curve of the rigid body model fitted to the experimental curve is shown in Supplementary Fig. 2c. According to the elongated conformation of VhaC (Fig. 1h), the PPC domain (magenta), connected by a flexible linker, extends outside the core region, self-consistent with the flexibility of VhaC observed in the Kratky plot (Supplementary Fig. 2d). Notably, the solution structure of VhaC is significantly different from that of the M9B collagenase ColH determined by SAXS analysis, which adopts a tapered shape with a swollen head and an elongated tail^37^.

### The triple-helical collagen recognition mechanism of CM

**Fig. 2:**
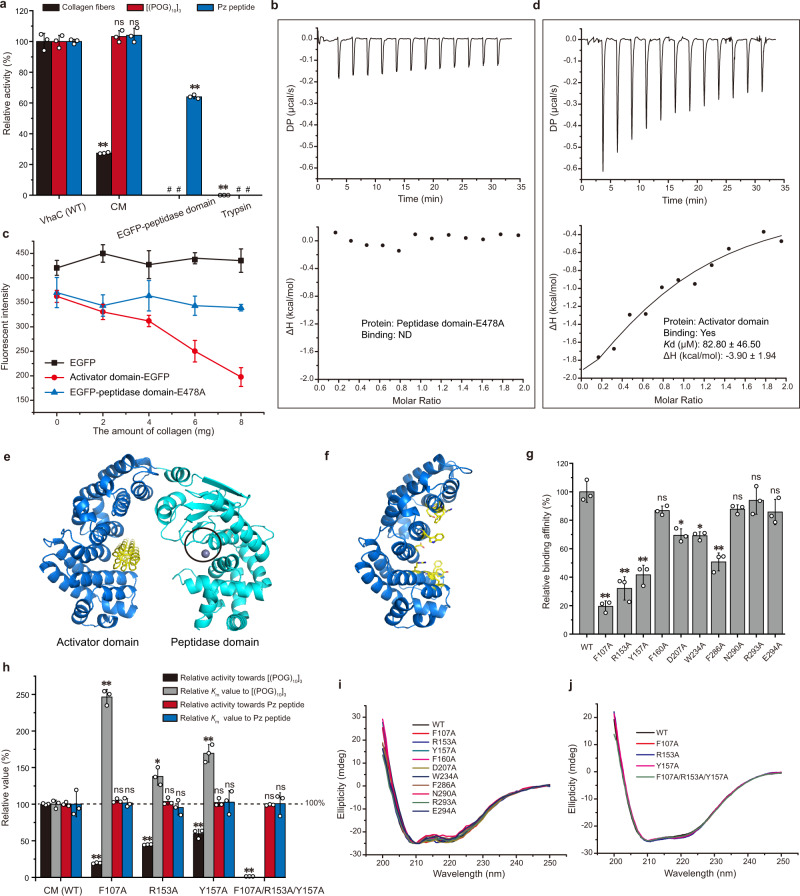
The triple-helical collagen recognition mechanism of CM. **a** The activities of VhaC, CM, and EGFP-peptidase domain towards collagen fibers, [(POG)_10_]_3_ and Pz peptide. The specific activities of VhaC (WT) towards collagen fibers, [(POG)_10_]_3_ and Pz peptide were taken as 100%. Trypsin was served as a negative control to show that collagen fibers were not denatured under the experimental conditions. **b** ITC analysis of the ability of peptidase domain-E478A to bind [(POG)_10_]_3_. Data representative of the results of triplicate experiments are shown. **c** Fluorescence analysis of the collagen fiber-binding ability of EGFP, activator domain-EGFP, and EGFP-peptidase domain-E478A. **d** ITC analysis of the [(POG)_10_]_3_-binding ability of the activator domain. Data representative of the results of triplicate experiments are shown. **e** Molecular docking of CM with THP (PDB: 1K6F). THP docked into the activator domain is shown in yellow. The catalytic center of the peptidase domain is circled. **f** The residues (yellow sticks) in the activator domain selected for site-directed mutation. **g** The collagen-binding ability of activator domain-EGFP (WT) and its mutants. **h** The activities and *K*_m_ values of CM and its mutants towards [(POG)_10_]_3_ and Pz peptide. The specific activities and *K*_m_ values of CM (WT) towards [(POG)_10_]_3_ and Pz peptide were taken as 100%, respectively. **i** CD spectra of activator domain-EGFP (WT) and its mutants. **j** CD spectra of CM (WT) and its mutants. The data shown in figures **i** and **j** are representatives of triplicate experiments. Data shown in figures **a**, **c**, **g**, and **h** are means ± standard deviations (SD) (*n* = 3 independent experiments). For comparison of the statistical differences between two groups, a two-tailed *t*-test was used in statistical analysis. * *p* < 0.01. ** *p* < 0.001. ns, no significant difference (*P* ≥ 0.01). # The significance was not compared due to the activity of the enzyme was undetectable. The *p*-values in all cases were compared with the corresponding wild type, and provided in the source data. Source data are provided as a Source Data file.

The triple-helical collagen recognition and degradation mechanisms of CM were then investigated. Firstly, the activities of VhaC, CM, and EGFP-peptidase domain (the peptidase domain fused to the enhanced green fluorescent protein, since the recombinant peptidase domain alone underwent severe autolysis during purification) against insoluble type I collagen fibers, triple-helical peptide (THP) [(POG)_10_]_3_, and 4-phenylazobenzyloixycarbonyl-Pro-Leu-Gly-Pro-o-Arg (Pz peptide) were compared. VhaC showed evident activity against all three substrates, whereas trypsin (control) showed negligible activity against collagen fibers and no activity against [(POG)_10_]_3_ or Pz peptide (Fig. 2a). The slight activity of trypsin on collagen fibers may be due to its degradation of proteoglycans or telopeptides within collagen fibers^38^. Compared to VhaC, CM showed approximately 100% activity against Pz peptide and [(POG)_10_]_3_ and only 27% activity against type I collagen fibers (Fig. 2a), EGFP-peptidase domain showed approximately 70% activity against Pz peptide but no activity against [(POG)_10_]_3_ or type I collagen fibers (Fig. 2a). Consistently, the inactive mutant of the peptidase domain, peptidase domain-E478A, had no detectable [(POG)_10_]_3_-binding ability (Fig. 2b) and the inactive mutant EGFP-peptidase domain-E478A, had little collagen fiber-binding ability (Fig. 2c). These results suggest that the activator domain plays an indispensable role in the degradation of triple-helical collagen and collagen fibers, likely involved in substrate binding. Indeed, isothermal titration calorimetry (ITC) measurements showed that the recombinant activator domain had [(POG)_10_]_3_-binding ability (Fig. 2d) and fluorescence detection showed that the activator domain-EGFP protein had collagen fiber-binding ability (Fig. 2c).

To investigate the triple-helical collagen-binding mode in CM, an attempt was made to obtain the crystal structure of the inactive mutant CM-E478A binding [(POG)_10_]_3_, but this failed. Instead, the structure of unbound-CM (the structure of CM with peptides P1 and P2 being removed) in complex with a THP (PDB: 1K6F) was modeled by molecular docking. According to the binding mode with the highest score, THP is bound to the potential substrate-recognition interface of the activator domain by hydrophobic interactions (Fig. 2e), away from the site responsible for peptide recognition in the catalytic center of the peptidase domain. This is consistent with the afore mentioned ITC data showing that THP [(POG)_10_]_3_ can bind to the activator domain (Fig. 2d) but not to the peptidase domain (Fig. 2b). The key amino acid residues in the activator domain for collagen binding were further investigated by site-directed mutagenesis. Ten residues (Phe107, Arg153, Tyr157, Phe160, Asp207, Trp234, Phe286, Asn290, Arg293, and Glu294) interacting with peptides P1 and P2 in the CM structure and/or with THP in the modeled complex structure were mutated to Ala (Fig. 2f), and the mutants were expressed as EGFP-fused proteins for detecting their collagen fiber-binding ability. These residues are strictly or highly conserved among M9 collagenases (Supplementary Fig. 3). Among the mutants, the collagen fiber-binding ability of mutants F107A, R153A, and Y157A was severely reduced compared with that of the wild type (Fig. 2g), indicating that these residues are likely the key residues involved in collagen binding. Consistently, single mutation of these amino acid residues (Phe107, Arg153, and Tyr157) in CM to Ala resulted in noticeable decrease in the activity of CM to [(POG)_10_]_3_ and an increase in its *K*_m_ value to [(POG)_10_]_3_ (Fig. 2h and Supplementary Fig. 4) and the triple mutant F107A/R153A/Y157A almost completely lost its enzymatic activity towards [(POG)_10_]_3_ (Fig. 2h). These results indicate that Phe107, Arg153 and Tyr157 are key residues involved in triple-helical collagen binding in the catalysis of CM. In contrast, these mutations (F107A, R153A, Y157A, and F107A/R153A/Y157A) had little effect on the enzymatic activity of CM toward Pz peptide and caused no change in the *K*_m_ value (Fig. 2h and Supplementary Fig. 4), suggesting that the activator domain in CM is not involved in the recognition of peptide substrates, consistent with the result that EGFP-peptidase domain had activity against Pz peptide (Fig. 2a). Circular dichroism (CD) spectroscopy assays showed that the secondary structures of all the site-directed mutants are similar to those of the wild types (Fig. 2i, j), suggesting that the mutations caused little structural alteration.

Together, these results indicate that VhaC adopts different strategies for the recognition of triple-helical collagen and peptide substrates. Triple-helical collagen is initially recognized by the activator domain, whereas peptide is directly recognized by the peptidase domain.

Since the above results are significantly different from the chew-and-digest model of ColG^22^, in which the triple-helical collagen is initially recognized by the peptidase domain, the activator domain, and the peptidase domain of ColG were expressed in *E. coli*, and the collagen-binding ability of the recombinant proteins was analyzed. Similarly, the activator domain of ColG showed evident binding ability towards collagen fibers (Supplementary Fig. 5a) and [(POG)_10_]_3_ (Supplementary Fig. 5b) but the peptidase domain did not (Supplementary Fig. 5a, c), suggesting that in ColG the activator domain rather than the peptidase domain, is likely responsible for the initial collagen recognition, just as in the case of VhaC.

### The catalytic mechanism of CM for triple-helical collagen

**Fig. 3:**
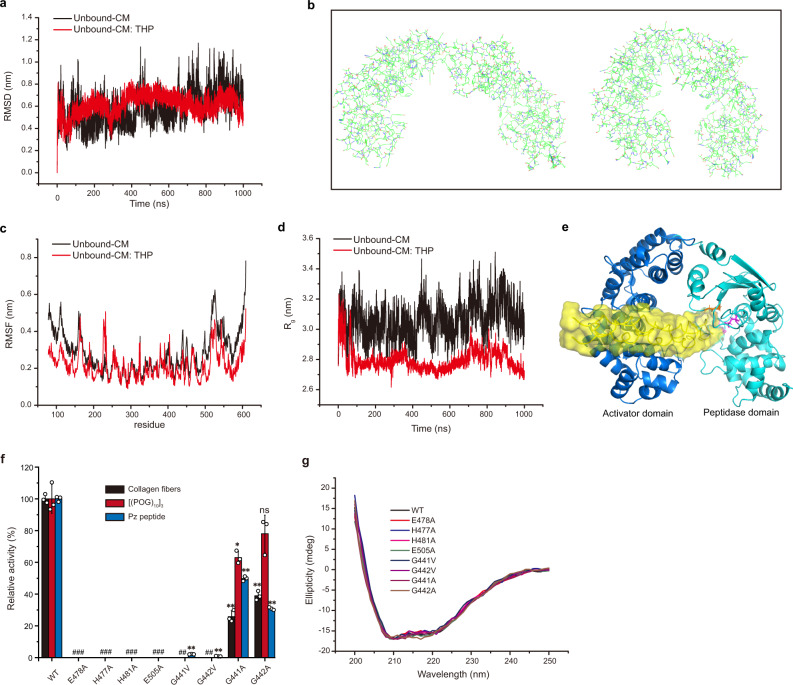
The catalytic mechanism of CM for triple-helical collagen. **a** RMSD of the backbone atoms of the unbound-CM and unbound-CM: THP complex structures. **b** Analysis of the conformational change of the first principal component in the unbound-CM MDS. **c** RMSF of the CM residues in the MDS of the unbound-CM and unbound-CM: THP structures. **d** Radius of gyration (*R*_g_) of the unbound-CM and unbound-CM: THP structures over simulated time. **e** The most populated cluster during the MDS of the unbound-CM: THP binary complex. The catalytic Glu478 (magenta) and double-Gly motif (orange) are labeled. The THP is indicated as surface and colored in yellow. **f** The activities of VhaC (WT) and its mutants towards type I collagen fibers, [(POG)_10_]_3_, and Pz peptide. The specific activities of VhaC towards collagen fibers, [(POG)_10_]_3_ and Pz peptide were taken as 100%. Data shown are mean ± SD (*n* = 3 independent experiments). For comparison of the statistical differences between two groups, a two-tailed *t*-test was used in statistical analysis. * *p* < 0.01. ** *p* < 0.001. ns, no significant difference (*P* ≥ 0.01). # The significance was not compared due to the activity of the enzyme was undetectable. The *p*-values in all cases were compared with the corresponding wild type, and provided in the source data. **g** CD spectra of VhaC (WT) and its mutants. The data shown are representatives of triplicate experiments. Source data are provided as a Source Data file.

Considering that triple-helical collagen preferentially binds to the activator domain rather than the peptidase domain, it can be speculated that the activator domain is responsible for collagen recognition while the peptidase domain is responsible for catalysis in CM. To investigate the catalytic mechanism of CM towards triple-helical collagen, unbound-CM and unbound-CM: THP binary complex were considered for performing molecular dynamics simulation (MDS) for 1000 ns. The RMSD profiles of the backbone atoms of the unbound-CM structure and its complex structure with THP show that all simulations generated stable trajectories (Fig. 3a), indicating that the systems reached the equilibrium state. Following equilibrium, unbound-CM showed continuous opening and closing changes (Supplementary Movie 1). Figure 3b shows the conformational opening and closing changes of the first principal component by principal component analysis, whose movement mode contributes more than 45% to the conformational change of CM. Cluster analysis also elucidates the open and closed states of unbound-CM (Supplementary Fig. 6). The root mean-square fluctuation (RMSF) of the N-terminal and C-terminal residues is greater than that of the middle residues (Fig. 3c), suggesting the open-closed movement of the activator domain and the peptidase domain. The radius of gyration (*R*_g_) of enzyme molecule (Fig. 3d) and the distances between the Cα atoms of the terminal residues in both domains (Supplementary Fig. 7) also show periodic changes. Supplementary Movie 2 shows the 1000 ns-MDS process of unbound-CM: THP binary complex. As the system gradually reached equilibrium, the THP bound to the activator domain approached the catalytic center of the peptidase domain with the conformation closing of CM. In addition, the RMSF of the N-terminal and C-terminal residues (Fig. 3c) and the *R*_g_ of the enzyme molecule (Fig. 3d) decrease compared with those in the unbound-CM MDS. These results potentially suggest that CM maintained an open-closed movement in the unbound-CM MDS, and adopted a closed state after binding to the triple-helical collagen in the unbound-CM: THP complex MDS. The most populated cluster in the MDS of the unbound-CM: THP complex is shown in Fig. 3e, and the distance between the catalytic residue and the nearest peptide bond of the THP in this closed state is approximately 6 Å. Since the peptidase domain had activity against the peptide substrate (Fig. 2a), an unwinding transition of triple helix molecule may occur, allowing a peptide chain and the catalytic residue to reach a distance for interaction (≤3 Å).

Further site-directed mutations also demonstrated the catalysis of the peptidase domain on collagen substrates. Glu478, which is strictly conserved among the M9 collagenases, is the general base (Supplementary Fig. 8). Mutation of Glu478 to Ala completely abolished the activity of VhaC towards type I collagen fibers, [(POG)_10_]_3_, or Pz peptide (Fig. 3f). Site-directed mutations of the ligands of the catalytic Zn^2+^ (His477, His481, and Glu505) to Ala also completely abolished the activity of VhaC towards these three substrates (Fig. 3f). The conserved double-Gly motif (Gly441 and Gly442) (Supplementary Fig. 8) is the S1′ peptide recognition site and its amides form a secondary oxyanion pocket that assists in substrate hydrolysis^22,39^. Mutations of Gly441 and Gly442 to Ala significantly reduced the activities of VhaC towards collagen fibers, [(POG)_10_]_3_ and Pz peptide. Furthermore, mutations of Gly441 and Gly442 to Val almost completely abolished the activity of VhaC against the three substrates (Fig. 3f), indicating that the increase of steric hindrance caused by side-chain affects the function of the amides in the double-Gly motif. CD spectroscopy analysis showed that all mutants have a similar secondary structure to that of the wild-type VhaC (Fig. 3g), suggesting that the activity loss of the mutants is caused by amino acid replacement rather than structural alteration. Together, these results help to identify the key residues involved in collagen catalysis in the peptidase domain of VhaC.

### Functional analysis of the PKD-like and the PPC domains

**Fig. 4:**
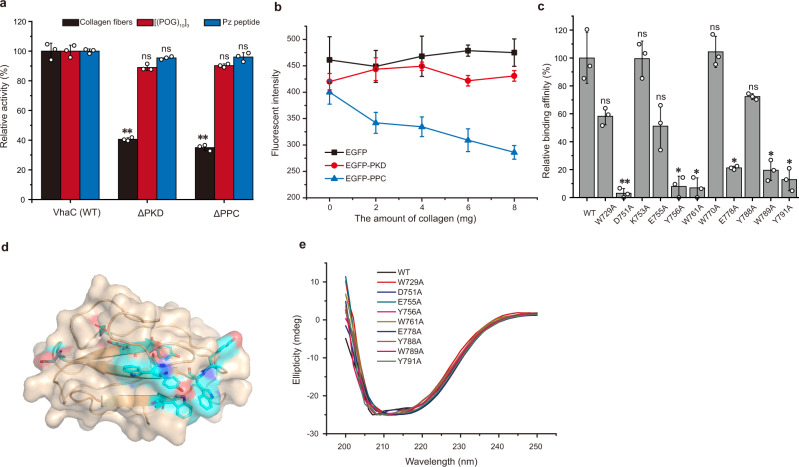
Functional analysis of the PKD-like and the PPC domains. **a** The activities of VhaC, ΔPKD, and ΔPPC towards collagen fibers, [(POG)_10_]_3_, and Pz peptide. The specific activities of VhaC (WT) towards type I collagen fibers, [(POG)_10_]_3_, and Pz peptide were taken as 100%. **b** Fluorescence analysis of the collagen-binding ability of EGFP, EGFP-PKD, and EGFP-PPC. **c** The collagen-binding ability of EGFP-PPC (WT) and its mutants. **d** Surface representation of the interface with collagen in the homology-modeled PPC domain. Residues involved in interacting with collagen are shown as cyan sticks. **e** CD spectra of EGFP-PPC (WT) and its mutants. The data shown are representatives of triplicate experiments. Data shown in figures **a–c** are means ± SD (*n* = 3 independent experiments). For comparison of the statistical differences between two groups, a two-tailed *t*-test was used in statistical analysis. * *p* < 0.01. ** *p* < 0.001. ns, no significant difference (*P* ≥ 0.01). The *p*-values in all cases were compared with the corresponding wild type, and provided in the source data. Source data are provided as a Source Data file.

As shown in Fig. 1a, VhaC contains two C-terminal accessory domains, the PKD-like domain, and the PPC domain. Compared to VhaC, CM had only 27% activity towards type I collagen fibers (Fig. 2a), suggesting that the two C-terminal accessory domains play an important role in the degradation of insoluble collagen fibers by VhaC. To reveal the functions of the PKD-like and the PPC domains, mutants ΔPKD (lacking only the PKD-like domain) and ΔPPC (lacking only the PPC domain) were constructed. Compared to VhaC, ΔPKD retained more than 88% activity towards Pz peptide or [(POG)_10_]_3_ but only 40% towards collagen fibers. ΔPPC retained more than 90% activity towards Pz peptide or [(POG)_10_]_3_ but only 35% towards collagen fibers (Fig. 4a). Therefore, the absence of either accessory domain severely affected the activity of VhaC towards collagen fibers. It has been reported that the PKD-like and the PPC domains of certain S8 and M4 proteases are able to bind and swell collagen to facilitate the enzymatic hydrolysis of collagen^40–42^. The PPC domains of some M9 collagenases have also been identified as CBD^32,43–46^. Therefore, the ability of the PKD-like domain and the PPC domain of VhaC to bind and swell collagen fibers was investigated. The EGFP-PKD protein had neither collagen-binding (Fig. 4b) nor collagen-swelling ability. Because the PKD-like domain is an intermediate domain in VhaC, its absence will affect the spatial arrangement of upstream (CM) and downstream (PPC) domains, thereby affecting the activity of the full-length enzyme against collagen fibers. Thus, it is likely that the PKD-like domain functions as a linker between the CM and the PPC domain.

The EGFP-PPC protein showed noticeable collagen-binding ability (Fig. 4b), but no collagen-swelling ability, suggesting that the PPC domain likely functions as a CBD. Moreover, based on the elongated conformation of VhaC determined by SAXS analysis, the PPC domain is connected to the core region by a flexible linker, suitable for expressing the collagen-binding function. The amino acid residues involved in binding collagen in the PPC domain were further investigated. Because aromatic residues and charged residues usually play key roles in insoluble collagen binding^40,47^, strictly or highly conserved aromatic residues (Trp729, Tyr756, Trp761, Trp770, Tyr788, Trp789, and Tyr791) and charged residues (Asp751, Lys753, Glu755, and Glu778) in the PPC domain were selected based on sequence alignment (Supplementary Fig. 9) and mutated to Ala, and all the mutants were expressed as EGFP fusion proteins. Fluorescence detection indicated that the collagen-binding ability of mutants W729A, D751A, E755A, Y756A, W761A, E778A, Y788A, W789A, and Y791A were reduced compared to that of the wild type EGFP-PPC (Fig. 4c), indicating that these residues may be essential for the PPC domain to bind to collagen. Coincidentally, all of these residues (except Tyr756) are located on one face of the homology-modeled PPC domain, with their side chains exposed to the solvent (Fig. 4d), and therefore, likely constitute the interface with collagen in the PPC domain, which, however, needs further study based on crystal structure. CD spectroscopy analysis showed that all mutants have a similar secondary structure to that of the wild-type EGFP-PPC (Fig. 4e), suggesting that the decreased collagen-binding ability of the mutants is caused by amino acid replacement rather than structural alteration.

### A model for collagen fibers degradation by VhaC

**Fig. 5:**
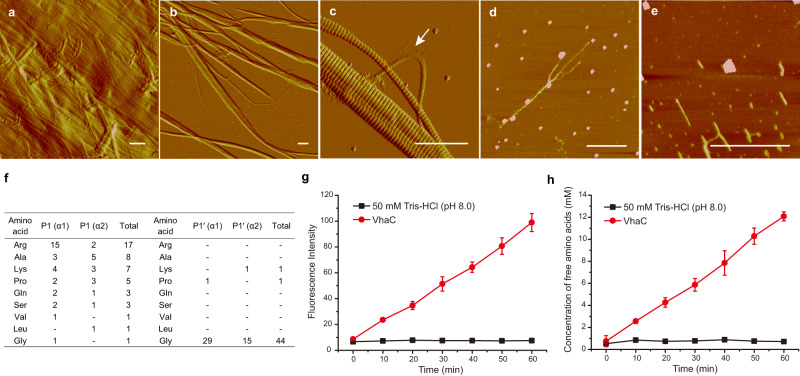
Degradation pattern of VhaC on type I collagen fibers. AFM observation of collagen fibers degradation by VhaC. Collagen fibers were treated with 50 mM Tris-HCl (pH 8.0) (**a**) or 0.1 μM VhaC (**b**–**e**) at 30 °C for 5 h with continuous stirring and then observed by AFM. Tight collagen fibers were cut into fibrils fragments (**b**) and the cleavage (*arrow*) was clearly observed (**c**). The broken fibrils fragments were further degraded into microfibrils (**d**) and tropocollagen fragments (**e**). **a–c** are peak force error images and **d**, **e** are height images. Bars, 1 μm. Representative AFM imaging was derived from at least three biologically independent preparations. **f** Residue frequency at P1 and P1′ sites in the tropocollagen triple-helical region degradation by VhaC. **g**, **h** Content of pyridinolines (**g**) and amino acids (**h**) released from collagen fibers by VhaC. Collagen fibers were treated with 50 mM Tris-HCl (pH 8.0) or 0.5 μM VhaC at 37 °C for 1 h with continuous stirring. Content of pyridinolines is indicated by the fluorescence intensity in the supernatant of the digested mixture detected on an FP-6500 spectrofluorometer (Jasco, Japan) at an excitation wavelength of 325 nm and an emission wavelength of 400 nm. Data shown in figures **g** and **h** are means ± SD (*n* = 3 independent experiments). Source data are provided as a Source Data file.

*Vibrio* collagenase is one of the few enzymes capable of digesting native collagen with hierarchical structure. In addition to studying the mechanism of VhaC for triple-helical collagen recognition and catalysis, its degradation pattern on insoluble type I collagen fibers was also investigated. Compared with the compact structure of collagen fibers treated with 50 mM Tris-HCl (pH 8.0) at 30 °C for 5 h (Fig. 5a), collagen fibers treated with 0.1 μM VhaC were cut into fibril fragments (Fig. 5b) and the cleavage was clearly observed on the surface of the released fibril fragments (Fig. 5c) using atomic force microscopy (AFM). The broken fibril fragments were further digested into microfibrils (Fig. 5d) and tropocollagen fragments (Fig. 5e). Further nano LC-MS analysis indicated that VhaC has multiple cleavage sites on the tropocollagen α1 and α2 chains (Supplementary Table 4 and Supplementary Fig. 10). The P1 position is often occupied by Arg, Ala, Lys, or Pro, and Gly dominates the P1′ position, indicating that VhaC prefers to cleave the Y-Gly bonds in the repeating Gly-X-Y triplets in the tropocollagen peptide chains (Fig. 5f). It is worth noting that nearly 93% of the separated peptides are located on the C-telopeptide of tropocollagen (Supplementary Table 4), leading to an extremely high frequency of the enzyme cleavage sites in this region (Supplementary Fig. 10). The C-telopeptide is non-helical and exposed on the fibril surface^3^, thereby vulnerable for collagenase assaults^4^. The high frequency of cleavage sites on the non-helical C-telopeptide indicates that it is the most likely region on tropocollagen that VhaC first assaults. This is supported by the biochemical results showing that the amount of pyridinolines released from VhaC-treated type I collagen fibers increased with treatment time (Fig. 5g). Because pyridinolines are involved in the covalent cross-linking between telopeptides and the helical part of adjacent collagen molecules^48,49^, the release of pyridinolines suggests that the telopeptides within collagen fibrils are degraded, leading to the dissociation of microfibrils fragments and tropocollagen fragments, as observed in Fig. 5d and Fig. 5e. Further degradation of the released tropocollagen fragments resulted in the release of multiple peptides (Supplementary Table 4) and amino acids (Fig. 5h). In contrast, compared with VhaC, trypsin, as a control, released less pyridinolines (Supplementary Fig. 11a) and slight free amino acids (Supplementary Fig. 11b) from collagen fibers, because trypsin is capable of digesting the non-helical telopeptides and proteoglycans in collagen fibers, but cannot degrade the triple helix structure of tropocollagen^38^.

**Fig. 6:**
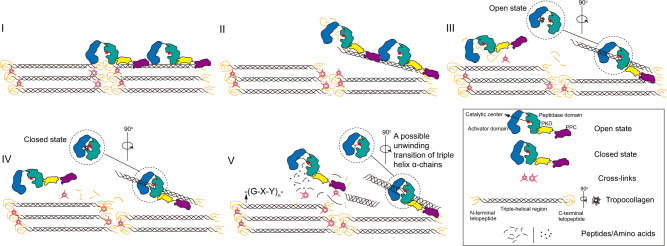
Schematic model of collagen fibers degradation by VhaC. Collagen fibers degradation by VhaC is processed in five steps: (I) VhaC molecules bind on collagen fibers via their PPC domains; (II) VhaC initiates the enzymatic hydrolysis of the C-telopeptide; (III) Triple-helical tropocollagen fragment enters the catalytic cavity and is recognized by the activator domain in the open state; (IV) Tropocollagen fragment is close to the catalytic center of the peptidase domain to initiate catalysis via conformational closing change of CM; (V) Tropocollagen fragments are subsequently unwound and progressively cleaved into peptides and amino acids by VhaC mainly at the Y-Gly bonds in the repeating Gly-X-Y triplets.

Based on all the above data, a stepwise collagenolysis model of the *Vibrio* collagenase VhaC was proposed in five main steps (Fig. 6): (I) VhaC molecules bind on collagen fibers via their PPC domains; (II) VhaC molecules then initiate the enzymatic hydrolysis of the C-telopeptide exposed on the fibril surface. With the degradation of C-telopeptide, the compact structure of the collagen fibrils disintegrates and triple-helical tropocollagen fragments are dissociated from the fibrils; (III) The released triple-helical tropocollagen fragment enters the open catalytic cavity and is recognized by the activator domain; (IV) The catalytic cavity is closed, making the tropocollagen fragment close to the catalytic center of the peptidase domain to initiate catalysis; (V) Tropocollagen fragments are subsequently unwound and progressively cleaved into peptides and amino acids via preferential attacks on the Y-Gly bonds in the repeating Gly-X-Y triplets.

## Discussion

Although many bacterial collagenases of the M9 family have been identified, structural information on the M9 bacterial collagenases is still limited. For *Vibrio* collagenases of the M9A subfamily, neither a domain nor an intact enzyme has three-dimensional structure information. VhaC from *Vibrio harveyi* VHJR7 is a true *Vibrio* collagenase composed of a CM containing an activator domain and a peptidase domain, a PKD-like domain, and a PPC domain. In this study, the crystal structure of the CM was solved, it having a more contracted saddle-shaped architecture and lacking the catalytic helper subdomain, when compared to that of *Clostridium* collagenase ColG, the only CM structure in the M9 family^22^. Moreover, the overall conformation and interdomain arrangement of the full-length VhaC in solution was analyzed by SAXS. The side-by-side arrangement of the CM, the PKD-like domain, and the PPC domain results in a long and flat overall conformation in solution, which is significantly different from the tapered form of the *Clostridium* collagenase, ColH (the only structural conformation in solution of an intact M9 collagenase determined by SAXS). For ColH, the compact arrangement of the CM and the PKD-like domain 1 constitutes its swollen head, and the PKD-like domain 2 and the PPC domain extending to the C-terminus form its tapered tail^37^. Among MMPs, the structure of MMP-1 and its complex with triple-helical collagen have been extensively studied by X-ray diffraction^12,50^ and nuclear magnetic resonance (NMR)^17^. The mature enzyme structure of MMP-1 shows a compact arrangement of the catalytic (CAT) domain and the C-terminal hemopexin (HPX)-like domain connected by a linker^12^, and the catalytic and structural Zn^2+^ ions play an important role in the structural stability and the dynamics of the MMP-1: THP complex^51^. There is no sequence or structural similarity between MMP-1 and VhaC.

Based on structural and biochemical analyses, the function of each domain of VhaC in collagenolysis was investigated and the mechanism of VhaC for triple-helical collagen recognition and catalysis was proposed. Presently, our understanding of collagen recognition and degradation by M9 collagenases largely comes from the chew-and-digest model of the *Clostridium* collagenase ColG proposed by Eckhard et al.^22^. In this model, the CM of ColG composed of the activator domain and the peptidase domain is suggested to switch between open and closed states for collagen hydrolysis. In the open state, triple-helical collagen initially docks to the peptidase domain. Followed by closing of the CM, triple-helical collagen is able to interact with both the activator domain and the peptidase domain. Subsequently, an entropy-driven unwinding of triple-helical collagen occurs, and the cleavage of the peptide chain follows^22^. Based on this model, only in the closed state can the activator domain interact with the triple-helical collagen bound to the peptidase domain and this assists the peptidase domain to complete the substrate unwinding^22^. In this study, molecular docking on the CM of *Vibrio* collagenase VhaC with a THP (PDB: 1K6F) suggested that triple-helical collagen is bound to the activator domain by hydrophobic interactions, but not the peptidase domain. Consistently, biochemical analysis showed that the activator domain of VhaC had binding ability to both THP [(POG)_10_]_3_ and collagen fibers, but its peptidase domain had neither. Moreover, it was also found that the activator domain of ColG showed evident binding ability towards [(POG)_10_]_3_ and collagen fibers, but its peptidase domain did not. Therefore, during collagen degradation by the M9 bacterial collagenases, such as VhaC and ColG, it is most likely that the activator domain is responsible for the initial recognition of triple-helical collagen, rather than the peptidase domain. This is different from the peptide recognition mode of the M9 collagenases. Both the results of this study and those reported on the M9B collagenases suggest that, for peptide degradation, the peptidase domain of the M9 collagenases alone can finish the recognition and catalysis of peptides^22,23^. However, for collagen degradation, the initial recognition of triple-helical collagen by the activator domain is indispensable.

Mammalian collagenase MMP-1 initially hydrolyzes the C-telopeptide on the outer edge of the collagen fibril to expose the cleavage site and permit interactions of the enzyme with the adjacent tropocollagen triple helix^4^. Similarly, *Vibrio* collagenase VhaC is likely to initially attack the C-telopeptide region to release tropocollagen fragments for further hydrolysis. However, the recognition and catalytic mechanisms of MMP-1 and VhaC on the triple-helical collagen monomer are significantly different. In addition to the CAT domain, the hydrolysis of triple-helical collagen by MMP-1 requires the exosite interaction of the HPX domain with the substrate^13,50^ and the interdomain flexibility modulated by the linker^17,18,52^. MMP-1 exhibits an equilibrium between open and closed conformations in solution^14–16^, and the binding of triple-helical collagen in the open conformation is productive^17,53^. MMP-1 in the open state binds on the triple-helical collagen via the HPX domain and the guidance of the flexible linker positions the CAT domain toward the intertwined cleavage site^17^. Back-rotation of the CAT and HPX domains to the closed conformation leads to the local unwinding of the triple helix, allowing a single-chain to enter the catalytic cleft and be cleaved^17^. The unwinding of triple-helical collagen (diameter, ~15 Å), which is stimulated by heat and compensated by entropy^54^, is a prerequisite for a single chain to enter the catalytic cleft (~5 Å) of MMP-1. In contrast, the hydrolysis of triple-helical collagen by VhaC is performed only by its CM that easily accommodates a triple-helical collagen monomer in its large catalytic cavity (~40 Å) and either the PKD-like domain or the PPC domain is not necessary. The activator domain in the CM initially binds the triple-helical collagen and the subsequent closing movement of the CM suggested by MDS studies enables the triple helix close to the catalytic center of the peptidase domain for hydrolysis. During this process, a local unwinding of the triple-helical collagen may occur. Eckhard and coworkers speculated that the final hydrolysis of triple-helical collagen by the CM of *Clostridium* collagenase ColG depends on an entropy-driven unwinding and the mechanical energy may come from the release of stored ordered water for substrate hydrolysis^22^. Santra et al.^55,56^ reported that *Vibrio* collagenase VMC that contains only a CM is likely to cause unwinding and structural destabilization of the triple-helical collagen molecule via its C-terminal FAXWXXT motif prior to hydrolysis. The key residues in this motif are conserved in *Vibrio* collagenases, providing information for revealing the underlying triple helix unwinding mechanism of *Vibrio* collagenases. Even so, how the triple-helical collagen is unwound before hydrolysis in the CM of the M9 collagenase still needs further study.

*Vibrio* collagenase of the M9A subfamily has been identified as an important virulence factor due to its ability to digest native collagen of the ECM^24^. This study reveals the structure of a *Vibrio* collagenase, VhaC, and its integrated mechanism of collagen recognition and degradation. This information will be crucial for future development of *Vibrio* collagenase as a drug target. In addition, functional domains that effectively bind to collagen have broader biomedical and clinical applications, such as developing collagen-targeted therapy. For example, CBDs of *Clostridium* collagenases are tailored for collagen-targeted drug delivery by fusing to growth factors^57,58^. The activator domain of VhaC consisting of an array of tandem α-helices has such a potential for its ability to target and bind collagen, which may be a promising target for designing drug delivery models. Sequence alignment shows that the key residues responsible for collagen binding are highly conserved in the M9 family, suggesting that the activator domains of other M9 collagenases, just like that of ColG, may also have collagen-binding function and act as such collagen-binding moiety in the drug delivery model.

## Methods

### Materials

Fish collagen was extracted from codfish skin using the method described by Chen et al.^59^. Briefly, shredded codfish skins bought from Shandong Oriental Ocean Co., Ltd. (China) were soaked with 0.1 M NaOH for 1 d, and then were treated with acetone by stirring for 3 h. The samples were then washed with distilled water and dissolved in 0.5 M acetic acid for 3 d with continuous stirring. After centrifugation, NaCl powder was added to the supernatant to a final concentration of 0.5 M. The precipitated collagen was dialyzed against distilled water and then freeze-dried. Type I collagen fibers from bovine achilles tendon were purchased from Worthington Biochemical Co., (USA). Collagens of types II-V, gelatin, casein, and Pz peptide were purchased from Sigma–Aldrich (USA). Collagenous peptide (POG)_10_ was purchased from Peptide Institute, Inc (Japan). Trypsin was purchased from Shanghai aladdin Biochemical Technology Co., Ltd. (China).

### Gene synthesis, mutagenesis, and protein expression and purification

The genes encoding VhaC (WP_047516938.1) and ColG (D87215.1) were synthesized by the BGI Tech Solutions Co., Ltd. (China) and cloned into the pET-22b vector (Novagen, USA) for protein expression. Using the recombinant plasmid pET-22b-*vhaC* and pET-22b-*colG* as templates, truncation mutations of VhaC and ColG were generated by PCR amplification or overlapping extension PCR^60^. The gene encoding EGFP amplified from the vector pEGFP-N1 (Clontech, USA) was used to fuse with DNA fragments for expressing EGFP fusion proteins. Site-directed mutations were performed using a modified QuikChange kit (Agilent Technologies, USA). The plasmids and primers used in this study are listed in Supplementary Data 1 and Supplementary Data 2, respectively. All mutants were verified by DNA sequencing.

The *E. coli* strains used in this study are listed in Supplementary Data 1. All of the plasmids for recombinant protein expression were transformed into *E. coli* BL21 (DE3) which were induced by the addition of 0.35 mM isopropyl β-D-1-thiogalactopyranoside (IPTG) at 15 °C for 16 h in the Lysogeny-Broth (LB) medium. The selenomethionine (SeMet) derivative of CM was over-expressed in *E. coli* BL21 (DE3) under the induction of 0.35 mM IPTG in the M9 minimal medium supplemented with selenomethionine, lysine, valine, threonine, leucine, isoleucine, and phenylalanine^61^. After cultivation, *E. coli* cells were collected and disrupted by high-pressure cell cracker in the buffer containing 50 mM Tris-HCl (pH 8.0), 100 mM NaCl, and 0.5% glycerol. The recombinant proteins were first purified by affinity chromatography with nickel-nitrilotriacetic acid resin (GE Healthcare, USA) and then by anion exchange chromatography using a Source 15Q column (GE Healthcare, USA). The target fractions were further purified by gel filtration chromatography using a Superdex 200 column (GE Healthcare, USA) eluted in the buffer containing 10 mM Tris-HCl (pH 8.0) and 100 mM NaCl. Protein concentration was determined using a BCA protein assay kit (Thermo Scientific, USA). Sedimentation velocity of the purified VhaC was performed on an XL-I analytical ultracentrifuge (Beckman Coulter, USA) at a speed of 210,000 × *g*. The sedimentation coefficients and molecular weight were calculated by SEDFIT V14.4 f software.

### Biochemical characterization of VhaC

Fish collagen, collagens of types I-V, gelatin, and casein were used as substrates for the enzyme specificity assays. The enzyme activities against type I collagen (fish collagen) and gelatin were determined using the colorimetric ninhydrin method with L-leucine as the standard^47^. For collagens of types II-V, the mixture of 0.05 mL enzyme solution at appropriate concentration and 0.25 mg substrate was incubated at 37 °C in 50 mM Tris-HCl (pH 8.0) for 0.5 h with continuous stirring. According to the method used by Philominathan et al.^45^, collagenous peptide (POG)_10_ was incubated with 50 mM Tris-HCl (pH 8.0) at 4 °C for 24 h before used for enzyme assays. The mixture of 5 μL enzyme solution at appropriate concentration and 5 μL [(POG)_10_]_3_ solution (1000 μM) was incubated at 25 °C for 30 min, and then 50% (v/v) of 1.25 M trichloroacetic acid (TCA) was added into the mixture to stop the reaction. The released amino acids were quantified using the colorimetric ninhydrin method with L-leucine as the standard. One unit of enzyme activity is defined as the amount of enzyme that releases 1 μmol leucine from types I-V collagens (fish collagen) in 1 h or [(POG)_10_]_3_ (gelatin) in 1 min. The activity toward casein was determined at 40 °C using the Folin-Ciocalteu method^62^. The activity toward Pz peptide was measured at 40 °C with the method described by Wuensch and Heidrich^63^ with modifications. Briefly, the mixture of 50 μL enzyme solution at appropriate concentration and 50 μL Pz peptide solution (1 mg/ml) was incubated at 40 °C for 30 min, and then 100 μL 1.25% (w/v) citric acid was added into the mixture to stop the reaction. After an addition of 1 mL ethyl acetate, the sample was mixed and centrifuged at 15,000 × *g* for 10 min. The absorbance of the upper phase was determined at 320 nm. One unit of enzyme activity is defined as the enzyme amount that increases 0.1 unit of absorbance at 320 nm per min. With type I collagen as the substrate, the optimum temperature for VhaC activity was determined in a range of 0-70 °C at pH 8.0. The optimum pH for VhaC activity was determined at 37 °C in Britton-Robinson buffers ranging from pH 3.0 to pH 12.0. Enzyme kinetic assays of wild-type CM and its mutants against [(POG)_10_]_3_ and Pz peptide were determined by nonlinear analysis. The initial rates were determined with 0 to 1500 μΜ [(POG)_10_]_3_ and 0 to 10 mg/ml Pz peptide in 50 mM Tris-HCl (pH 8.0). The *K*_m_ values were calculated by the Michaelis-Menten equation using the OriginPro 8.5 software.

### Crystallization, data collection, structure determination, and refinement

The protein concentration of CM for crystallization was 10 mg/mL in the buffer containing 10 mM Tris-HCl (pH 8.0) and 100 mM NaCl. Crystals of CM were obtained at 4 °C using the sitting drop method in the buffer containing 0.2 M sodium acetate trihydrate, 0.1 M Tris-HCl (pH 8.5), and 30% (w/v) polyethylene glycol 4000 after two weeks. Crystals of SeMet-CM were obtained at 4 °C using the sitting drop method in the buffer containing 0.2 M calcium acetate, 0.1 M imidazole/HCl (pH 8.0), and 10% (w/v) polyethylene glycol 8000 after two weeks. All the x-ray diffraction data were collected on the BL18U1 beamline at Shanghai Synchrotron Radiation Facility. The initial diffraction data sets were processed by the HKL3000 program^64^. The structure of CM was determined by single-wavelength anomalous dispersion phasing using a selenomethionine derivative. Experimental phases were solved using AutoSol in the Phenix program^65^. Initial mode was built using Phenix program AutoBuild. Refinement of the structure of CM was done by Phenix program Refine and Coot^66^ alternately.

### SAXS measurement

SAXS data from solutions of VhaC were collected on the BL19U2 beamline at Shanghai Synchrotron Radiation Facility, equipped with a PILATUS 1 M detector (DECTRIS, Switzerland) (Supplementary Table 3). The enzyme solutions ranging from 0.5 to 9 mg/mL in 10 mM Tris-HCl (pH 8.0) containing 100 mM NaCl at 10 °C were loaded by a robotic system into a 1.5-mm quartz capillary and 20 independent 0.5 s exposures were collected. All SAXS data processing was accomplished using BioXTAS RAW^67^ and ATSAS 3.0.3 software package^68^. The scattering profile of the protein was obtained after subtracting the buffer profile. The forward scattering, *I*(0), and the radius of gyration, *R*_g_, were evaluated using the Guinier approximation. The maximum dimension, *D*_max_, and the interatomic distance distribution function (*P*(*r*)) were calculated using the program GNOM^69^. The Kratky plot was used to analyze for protein flexibility^70^. Ab initio modeling of the molecular envelope was calculated and optimized by the program DAMMIF^71^ and DAMMIN^72^. Rigid body model construction to the experimental scattering data was performed using CORAL^73^, using the individual domain models of VhaC. Except for the crystal structure of CM, the PKD-like domain model was prepared by SWISS-MODEL^74^, using the crystal structure of the PKD-like domain from ColG (PDB: 4TN9) as the template. For the model of the PPC domain, solution structure of the PPC domain derived from the M4 metalloprotease vEP (PDB: 2LUW) was used. The modeled structures lacked structural information of the linker region between the PKD-like and the PPC domains (Thr691 to Gly712). Therefore, a poly-glycine segment was involved in the rigid body modeling process using CORAL, replacing this missing linker. The theoretical scattering curve from the atomic model was calculated and compared with the experimental curve by CRYSOL^75^. Atomic model was docked into ab initio envelope with the program SUBCOMB^76^.

### Collagen binding and swelling assay and CD spectroscopy assay

Collagenous peptide (POG)_10_ was incubated in the buffer of 10 mM Tris-HCl (pH 8.0) containing 100 mM NaCl at 4 °C for 24 h before ITC measurement. Titrations were performed by adding 0.4 μL of [(POG)_10_]_3_ solution for the first injection and 3 μL for the following 12 injections to the protein solution with stirring using a MicroCal PEAQ-ITC system (Malvern, United Kingdom) at 25 °C. The concentrations of [(POG)_10_]_3_ were 1000 μM for the activator domain and the peptidase domain-E478A proteins (100 μM) and 262 μM for the ColG-activator domain and the ColG-peptidase domain-E524A proteins (150 μM). The experimental data were analyzed using Microcal PEAQ-ITC analysis software. For the binding of insoluble type I collagen fibers, the individual domains fused with EGFP at a concentration of 12.5 μM were incubated with various amounts of collagen fibers (0, 2, 4, 6, and 8 mg) in 50 mM Tris-HCl (pH 8.0) at 30 °C for 2 h with stirring. The free fluorescence intensity in the supernatant was detected on an FP-6500 spectrofluorometer (Jasco, Japan) at an excitation wavelength of 489 nm and an emission wavelength of 510 nm. The collagen-binding ability of fusion proteins and their site-directed mutants was determined with the free fluorescence intensity in the supernatant before and after incubation with 5 mg collagen fibers. Binding affinity (%)=(X-Y)/X*100%, where X and Y represent the free fluorescence intensity in the solution before and after incubation, respectively.

As for collagen-swelling assay, insoluble type I collagen fibers (5 mg) were incubated with 12.5 μM EGFP-PKD or EGFP-PPC in 1 mL 50 mM Tris-HCl (pH 8.0) at 30 °C for 12 h with continuous stirring. Then, the collagen-swelling effects of the two fusion proteins were checked based on visual inspection.

CD spectra of the purified VhaC and its mutants (0.5 mg/mL) in the buffer containing 10 mM Tris-HCl (pH 8.0) and 100 mM NaCl were recorded from 250 nm to 200 nm at a scanning rate of 200 nm/min with a bandwidth of 1 nm on a J1500 spectropolarimeter (Jasco, Japan) at 25 °C. JASCO Spectra Manager was used for data collection and analysis.

### Molecular dynamics simulation

Unbound-CM was constructed by manually removing peptide P1 and P2 from the structure of CM with PyMOL. The structure of a THP was obtained from the Protein Data Bank (ID: 1K6F). Unbound-CM and THP were subjected to Z-Dock (http://zdock.umassmed.edu/) to generate probable binding modes with default parameters. Unbound-CM and the most reasonable complex structure of unbound-CM with THP were subjected to the software package GROMACS 2019^77^ for a 1000-ns MDS, respectively, with the AMBER99SB-ILDN^78^ force field adopted. All simulations were performed under the NPT ensemble with periodic boundary conditions and a time step of 2 fs. The temperature of the system was kept at 298 K using the v-rescale method, and the pressure was kept at 1 bar using the Parrinello-Rahman method. According to the backbone-atom RMSD plot, trajectories that reached the equilibrium state (500-1000 ns) were used for further principal component and cluster analyses.

### The degradation pattern of VhaC on type I collagen fibers

AFM was used to observe the collagen fibers before and after enzymatic treatment. Insoluble bovine type I collagen fibers (5 mg) were treated with 0.1 μM VhaC or 50 mM Tris-HCl (pH 8.0) (control) at 30 °C for 5 h with continuous stirring. After treatment, the samples were centrifuged at 10,000 × *g*, rinsed with distilled water, and spread onto freshly cleaved mica. After drying in the air, imaging was carried out in ScanAsyst mode using a Multimode Nanoscope VIII AFM (Bruker AXS, Germany) with a J-type scanner. AFM imaging data were collected using Nanoscope v. 8.10. AFM data analysis was performed with NanoscopeAnalysis v. 1.40.

The cleavage sites of VhaC on tropocollagen α1 and α2 chains were analyzed by nano LC-MS. Insoluble type I collagen fibers (10 mg) were incubated with 0.2 μM VhaC in 50 mM Tris-HCl (pH 8.0) at 30 °C for 5 h with continuous stirring. The reaction was stopped by the addition of 1% trifluoroacetic acid and the digested product was analyzed by nano LC system (Eksigent Technologies, nano LC-Ultra 2D plus, USA) equipped with a high-resolution tandem mass spectroscopy system (Thermo Scientific, LTQ Orbitrap velos pro ETD, Germany). The sequences of released peptides were analyzed by Proteome Discoverer software 1.4 (Thermo Scientific, USA) with the Sequest HT search engine against ipi.BOVIN.v3.69.fasta database for data processing.

Thirty milligrams of insoluble type I collagen fibers were incubated with 0.5 μM VhaC or 50 mM Tris-HCl (pH 8.0) at 37 °C for 1 h with continuous stirring. Pyridinolines released from the covalent cross-links within collagen fibrils in the supernatant were detected on an FP-6500 spectrometer (Jasco, Japan) at an excitation wavelength of 325 nm and an emission wavelength of 400 nm^79^. The amino acid concentration in the supernatant released from collagen fibers was quantitatively analyzed by the colorimetric ninhydrin method with L-leucine as the standard. Collagen fibers (30 mg) incubated with 0.5 μM trypsin or 50 mM Tris-HCl (pH 7.5) at 37 °C for 1 h with continuous stirring were used as the control.

### Bioinformatics

SignalP 5.0 was used to predict the potential signal peptide of VhaC. NCBI Conserved Domain Database was used to analyze the conserved domain structure of VhaC. Multiple sequence alignment was performed with CLC Sequence Viewer 6 and edited with ESPript 3.0^80^.

### Statistics and reproducibility

Data analyses were carried out using Microsoft Excel 2019, OriginPro 8.5, and OriginPro 2018C. All data shown are means ± SD and were analyzed using two-tailed *t*-test where appropriate. For ITC analysis, CD spectroscopy analysis, AFM observation, and nano LC-MS analysis, similar results were obtained from three independent experiments. Detailed data analyses are described in the text.

### Reporting summary

Further information on research design is available in the Nature Research Reporting Summary linked to this article.

## Supplementary information

Supplementary Information

Description of Additional Supplementary Files

Supplementary Data 1

Supplementary Data 2

Supplementary Movie 1

Supplementary Movie 2

Reporting Summary

## Data Availability

All data supporting the findings of this study are available within the paper (and its Supplementary Information files). The structure data of CM have been deposited in the Protein Data Bank (PDB) database under accession code 7ESI. The SAXS data and models have been deposited in the Small Angle Scattering Biological Data Bank (SASBDB) under accession code SASDMU3. Other structure data used in this study are available in the Protein Data Bank (PDB) database under accession code 2Y50, 2Y6I, 1K6F, 4TN9, and 2LUW. The sequence data of VhaC and ColG used in this study are available in the GenBank database under accession codes WP_047516938.1 and D87215.1, respectively. A reporting summary for this article is available as a Supplementary Information file. Source data are provided with this paper.

## Source data

Source Data
