# Supplementary material for: Structure of Vibrio collagenase VhaC provides insight into the mechanism of bacterial collagenolysis
Source: Nat Commun. 2022 Jan 28;13:566. doi: 10.1038/s41467-022-28264-1 (PMC8799719; doi:10.1038/s41467-022-28264-1)
Supplement: Supplementary file 1 — Supplementary Information [file 41467_2022_28264_MOESM1_ESM.pdf]

Structure of *Vibrio* collagenase VhaC provides insight into the mechanism of bacterial collagenolysis

Wang et al

Supplementary figures

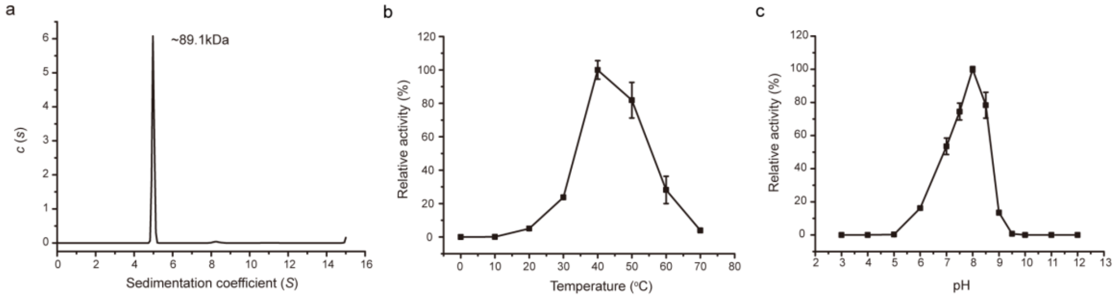

Supplementary Figure 1 | Characterization of VhaC using type I collagen as substrate. a, Sedimentation-velocity analytical ultracentrifugation (AUC) analysis of the form of VhaC in solution. The peak sedimentation coefficient and the calculated molecular weight for the VhaC protein are shown. b, Effect of temperature on VhaC activity. The specific activity of VhaC at 40°C was taken as 100%. c, Effect of pH on VhaC activity. The specific activity of VhaC at pH 8.0 was taken as 100%. Data shown in figures b and c are means ± standard deviations (SD) (n = 3 independent experiments). Source data are provided as a Source Data file.

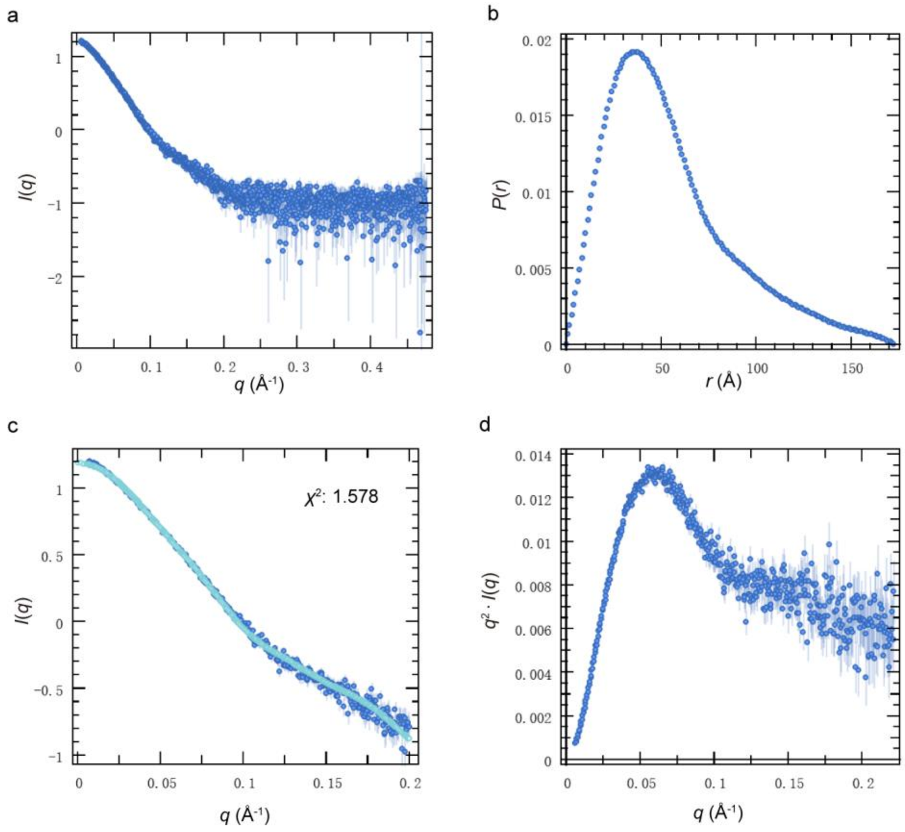

Supplementary Figure 2 | SAXS data of VhaC. a, Experimental scattering profile for VhaC. b, The *P*(*r*) distribution for VhaC. c, Fitting of the theoretical scattering curve from the rigid body model and the experimental curve of VhaC. d, The Kratky plot for VhaC. The figures were generated using PRIMUS in the ATSAS 3.0.3 software package. Source data are provided as a Source Data file.

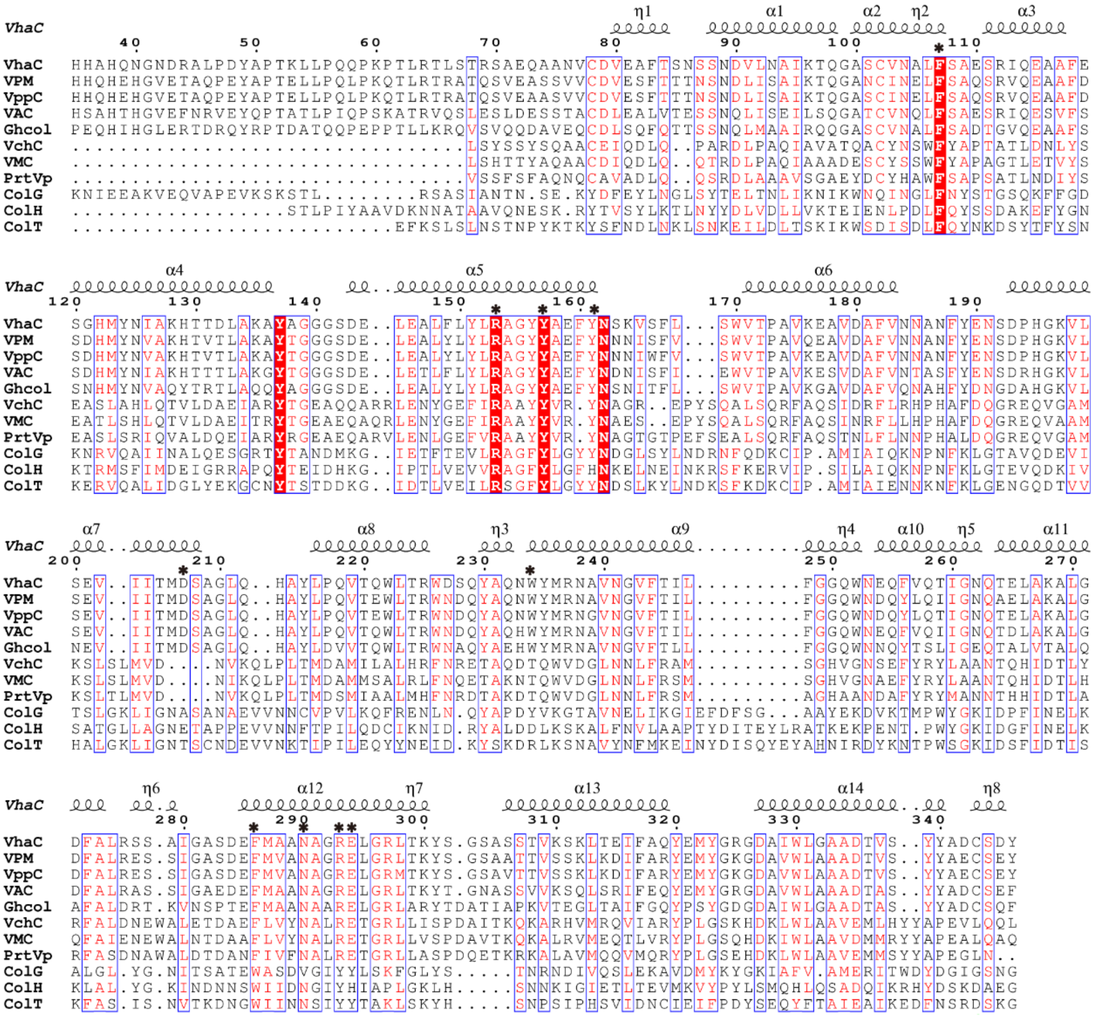

Supplementary Figure 3 | Multiple sequence alignment of the activator domains of the characterized M9 metalloproteases. Metalloproteases VhaC (GenBank accession no. WP_047516938.1), VPM (ABF19104.1), VppC (AAG59883.1), VAC (CAA44501.1), Ghcol (BAK39964.1), VchC (AAF94801.1) VMC (AAC23708.1), and PrtVp (CAA86734.1) are from the M9A subfamily, while ColG (D87215.1), ColH (D29981.1), and ColT (WP_011100838.1) are from the M9B subfamily. Secondary structures and amino acid numbering for VhaC are shown above the alignment. Amino acid residues indicated with “*” are those chosen for site-directed mutations to determine the key residues for collagen binding in the activator domain of VhaC.

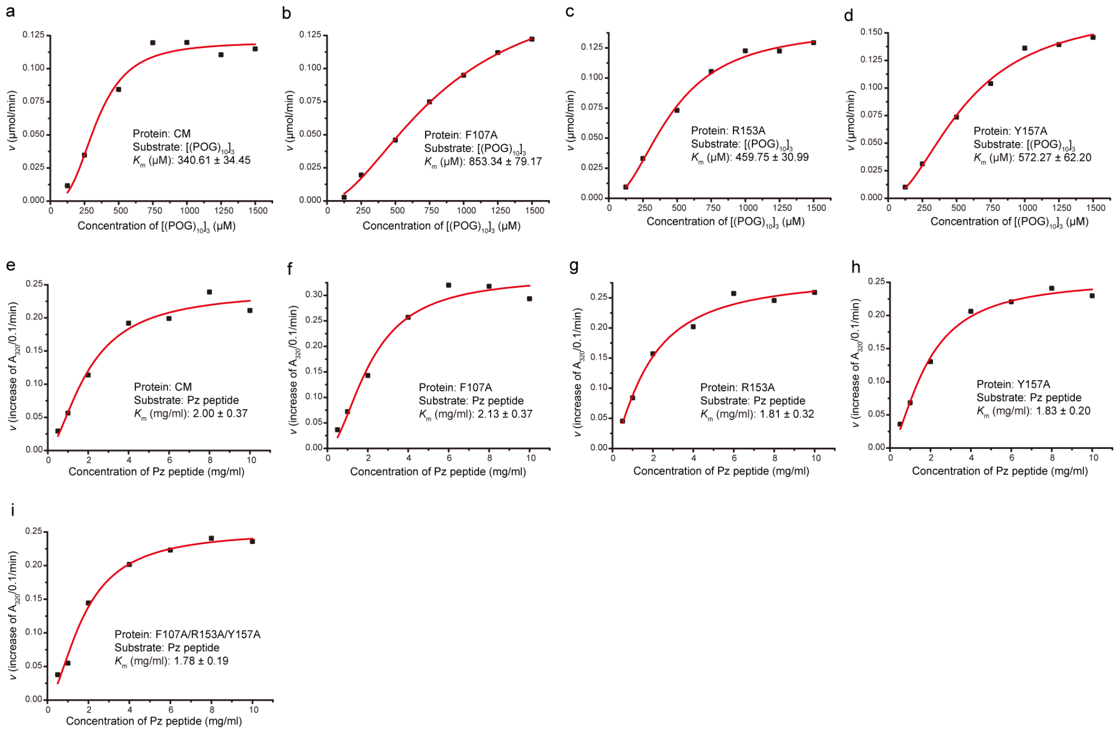

Supplementary Figure 4 | Non-linear fit curves for the hydrolysis of $[(POG)_{10}]_3$ and Pz peptide by CM and its mutants. These assays were performed in 50 mM Tris-HCl (pH 8.0) at 25°C for $[(POG)_{10}]_3$ and 40°C for Pz peptide. The K_m values were calculated by non-linear regression fit directly to the Michaelis-Menten equation using the OriginPro 8.5 software. a, Non-linear fit curve for the hydrolysis of $[(POG)_{10}]_3$ by CM. b, Non-linear fit curve for the hydrolysis of $[(POG)_{10}]_3$ by mutant F107A. c, Non-linear fit curve for the hydrolysis of $[(POG)_{10}]_3$ by mutant R153A. d, Non-linear fit curve for the hydrolysis of $[(POG)_{10}]_3$ by mutant Y157A. e, Non-linear fit curve for the hydrolysis of Pz peptide by CM. f, Non-linear fit curve for the hydrolysis of Pz peptide by mutant F107A. g, Non-linear fit curve for the hydrolysis of Pz peptide by mutant R153A. h, Non-linear fit curve for the hydrolysis of Pz peptide by mutant Y157A. i, Non-linear fit curve for the hydrolysis of Pz peptide by mutant F107A/R153A/Y157A. All data shown are means ± SD (n = 3 independent

experiments). Source data are provided as a Source Data file.

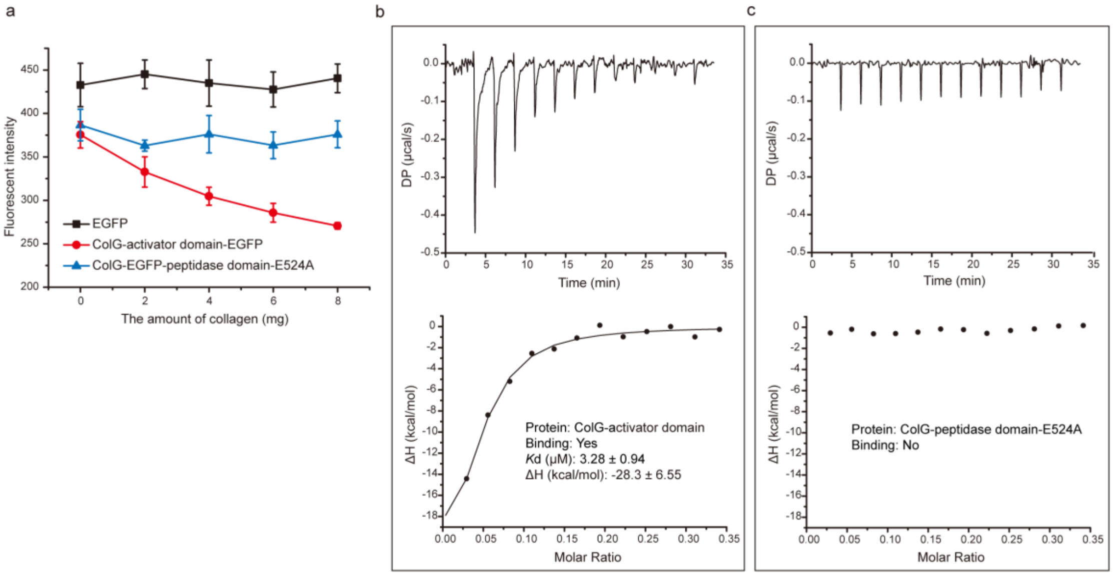

Supplementary Figure 5 | Analysis of the ability of the activator domain and the peptidase domain of ColG to bind collagen fibers and [(POG)$_{10}$]$_3$. a, Fluorescence analysis of the collagen fiber-binding ability of EGFP, ColG-activator domain-EGFP, and ColG-EGFP-peptidase domain-E524A. Data shown are mean ± SD (n = 3 independent experiments). b, ITC analysis of the [(POG)$_{10}$]$_3$-binding ability of ColG-activator domain. c, ITC analysis of the ability of ColG-peptidase domain-E524A to bind [(POG)$_{10}$]$_3$. Data representative of the results of triplicate experiments are shown. Source data are provided as a Source Data file.

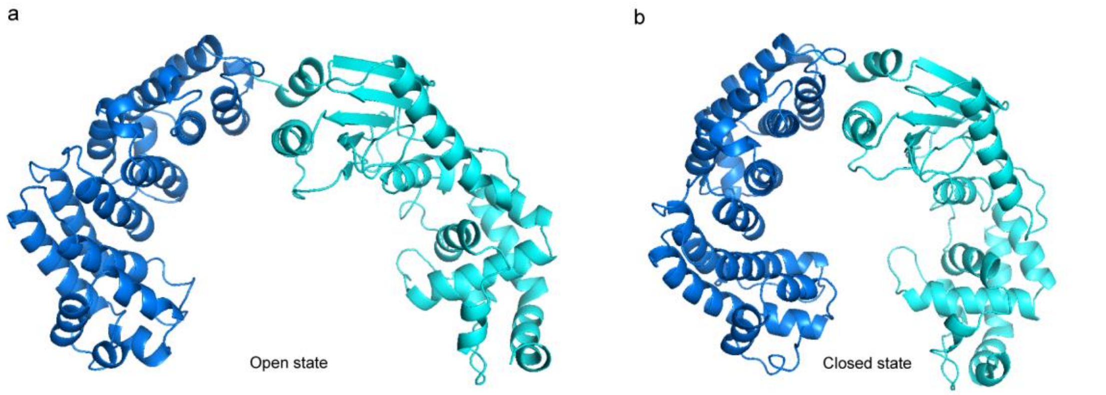

Supplementary Figure 6 | Cluster analysis of the open and close states of unbound-CM. The enzyme conformations obtained by cluster analysis show the open (a) and closed (b) states of unbound-CM.

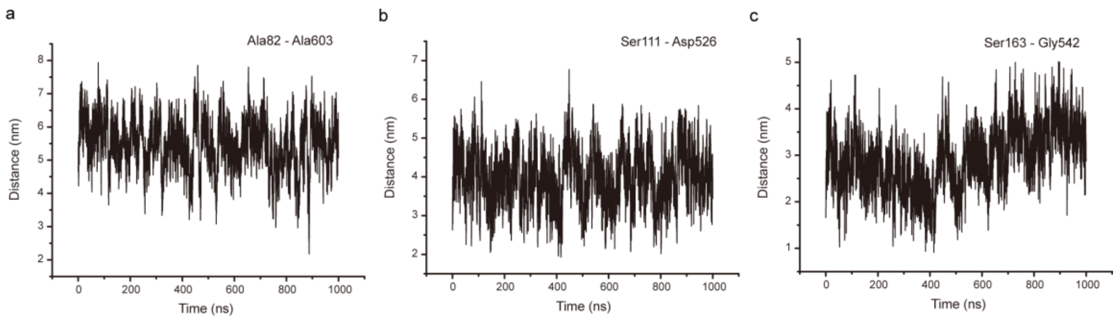

Supplementary Figure 7 | The distances between the Cα atoms of several pairs of terminal residues in the activator and the peptidase domains over the molecular dynamics trajectory. a, The distance between the Cα atoms of residues Ala82 and Ala603. b, The distance between the Cα atoms of residues Ser111 and Asp526. c, The distance between the Cα atoms of residues Ser163 and Gly542.

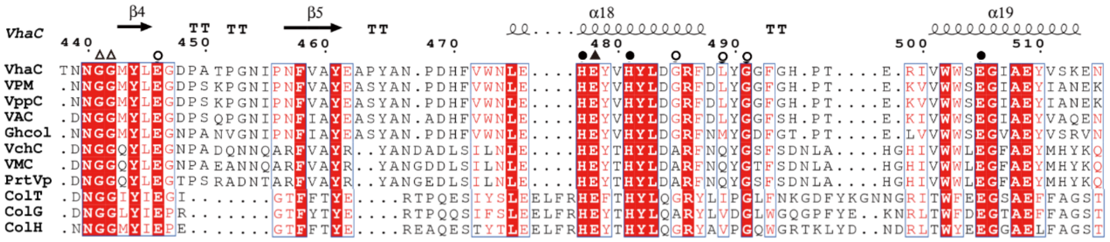

Supplementary Figure 8 | Sequence alignment of the catalytic centers of the characterized M9 metalloproteases. Metalloproteases VhaC (WP_047516938.1), VPM (ABF19104.1), VppC (AAG59883.1), VAC (CAA44501.1), Ghcol (BAK39964.1), VchC (AAF94801.1) VMC (AAC23708.1), and PrtVp (CAA86734.1) are from the M9A subfamily, while ColT (WP_011100838.1), ColG (D87215.1), and ColH (D29981.1) are from the M9B subfamily. Secondary structures and amino acid numbering for VhaC are shown above the alignment. Solid circles indicate residues involved in Zn^{2+} binding, open circles indicate residues involved in Ca^{2+} binding, the double Gly-motif is marked by open triangles and the catalytic residue Glu478 is marked by a solid triangle.

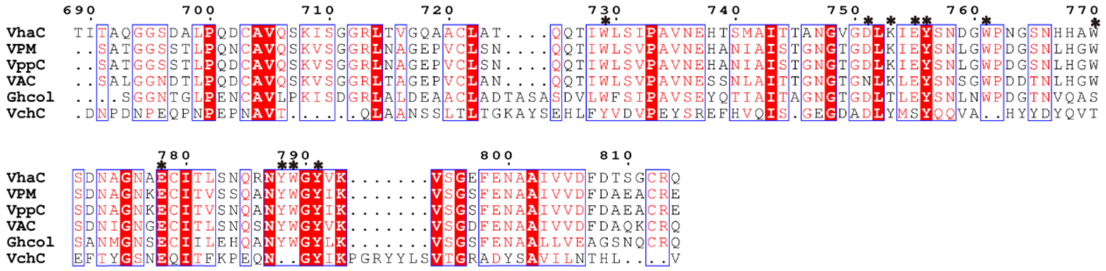

Supplementary Figure 9 | Sequence alignment of the PPC domains of the characterized M9A metalloproteases. The amino acid sequences are from VhaC (WP_047516938.1), VPM (ABF19104.1), VppC (AAG59883.1), VAC (CAA44501.1), Ghcol (BAK39964.1), and VchC (AAF94801.1). The amino acid numbering of VhaC is shown above the alignment. Amino acid residues indicated

with “*” are those chosen for site-directed mutations to determine the key residues for collagen binding in the PPC domain.

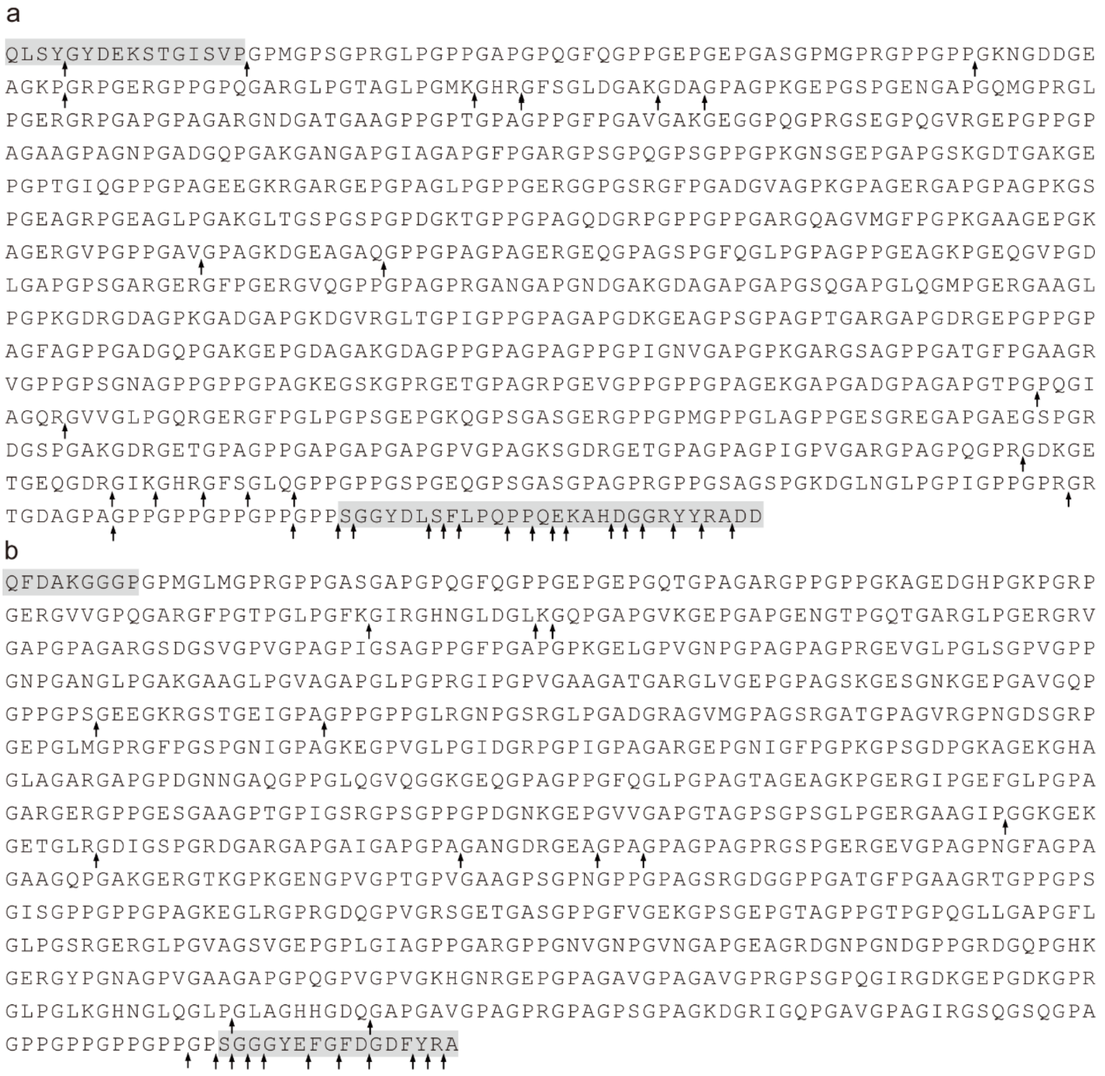

Supplementary Figure 10 | The cleavage sites of VhaC on tropocollagen α1 (a) and α2 (b) chains deduced based on the sequences of the released peptides shown in Supplementary Table 4. The N- and C-terminal telopeptides are indicated by *shadows*. The cleavage sites are indicated by *arrows*.

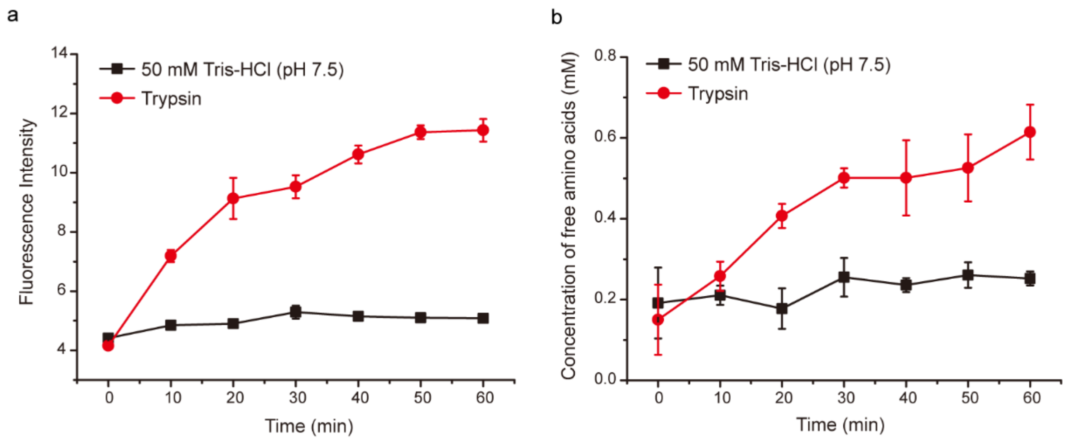

Supplementary Figure 11 | Analysis of pyridinolines (a) and amino acids (b) released from collagen fibers by trypsin. Collagen fibers (30 mg) were incubated with 50 mM Tris-HCl (pH 7.5) or 0.5 μM trypsin at 37°C for 1 h with continuous stirring. Content of pyridinolines in the supernatant of the digested mixture was detected on a FP-6500 spectrofluorometer (Jasco, Japan) at an excitation wavelength of 325 nm and an emission wavelength of 400 nm. Concentrations of amino acids in the supernatant of the digested mixture were detected at the indicated digestion times by the colorimetric ninhydrin method with L-leucine as the standard. All data shown are means ± SD (n = 3 independent experiments). Source data are provided as a Source Data file.

Supplementary tables

Supplementary Table 1 | Substrate specificity of VhaC.

**Substrate**	**Activity[a] (U/mg)**
Fish collagen fibers	1281.69 ± 70.12
Type I collagen from bovine achilles tendon	281.07 ± 15.13
Type II collagen from bovine nasal septum	378.66 ± 37.06
Type III collagen from human placenta	374.04 ± 51.31
Type IV collagen from human placenta	252.95 ± 29.34
Type V collagen from human placenta	32.45 ± 4.31
Gelatin	86.59 ± 3.82
Casein	ND[b]

[a]The specific activity of VhaC toward each substrate was measured in 50 mM Tris-HCl (pH 8.0). All data shown are means ± SD (n = 3 independent experiments). Source data are provided as a Source Data file.

[b]ND means that the enzyme activity was not detectable.

Supplementary Table 2 | Diffraction data and refinement statistics of CM and SeMet-CM.

**Parameters**	**CM**	**SeMet-CM**
Data collection		
Space group	$P\ 2_1$	$P\ 2_1$

Wavelength (Å)	0.9792	0.9792
Unit cell parameters	a=74.444 Å	a=74.139 Å
	b=50.068 Å	b=49.833 Å
	c=90.681 Å	c= 91.447 Å
	α=90°	α=90°
	β=104.72°	β=104.879°
	γ=90°	γ=90°
Resolution range (Å)	42.56-1.80 (1.87-1.80)	40.91-1.80 (1.86-1.80)
Unique reflections	59569 (5891)	60212 (5972)
Redundancy	3.4 (3.5)	6.7 (6.3)
Completeness (%)	99.1 (99.8)	99.96 (99.97)
R_{meas}	0.061 (0.164)	0.101 (0.543)
$R_{p.i.m}$	0.033 (0.087)	0.039 (0.215)
Mean $I/\sigma(I)$	23.95 (8.87)	29.50 (3.00)
Refinement statistics		
R_{work}[a]	0.161 (0.186)	
R_{free}	0.188 (0.221)	
Average B-factor (Å^2)	21.67	
Protein	19.94	
Water	31.35	
Ligands	16.13	
R.m.s.d. from ideal geometry		

R.m.s.d. length (Å)	0.007
R.m.s.d. angles (°)	0.76
Ramachandran Plot (%)	
Favored	97.64
Allowed	2.36
Outliers	0

Values in parentheses indicate the highest resolution bin

[a]$R_{work}=\sum_{hkl}||F_{obs}| - |F_{calc}||/\sum_{hkl} (F_{obs})$

Supplementary Table 3 | SAXS parameters of VhaC.

**Parameters**	
Data Collection	
Beam line	SSRF (Shanghai, China) BL19U2
Wavelength (Å)	0.923
Detector	Pilatus 1M
q range (Å $^{-1}$)	0.009-0.45
Exposure time (s)	0.5 s for 20 frames
Protein concentration (mg/ml)	0.5 to 9 mg/mL
Temperature (°C)	10
Structural Parameters	
$I(0)$ (cm^{-1}) from Guinier fit	15.65 ± 0.06
R_g (Å) from Guinier fit	42.58 ± 0.32

I(0) (cm^{-1}) from *P*(*r*)	15.77 ± 0.06
R_g (Å) from *P*(*r*)	44.82 ± 0.35
D_{max} (Å) from *P*(*r*)	176
Modeling	
DAMMIN χ^2	1.185
DAMMIN Ensemble Resolution	39 ± 3
DAMMIN NSD	0.634 ± 0.055
Software Employed	
Primary data Processing	RAW
P(*r*)	GNOM
Ab initio shape analysis	DAMMIF
Rigid body modeling	CORAL
SAXS Profile computation	CRYSOL
Molecular Visualization	PyMOL

Supplementary Table 4 | Peptides released from type I collagen fibers by VhaC[a].

Region	MH^+ [Da]	PSMs	Peptide sequence	Chain	Position
N-telopeptide	1252.60	1	Y.GYDEKSTGISVP.G	α-1	166 - 177
C-telopeptide	862.39	2	P.GPPSGGYDL.S	α-1	1189 - 1197
C-telopeptide	949.42	1	P.GPPSGGYDLS.F	α-1	1189 - 1198
C-telopeptide	1096.49	1	P.GPPSGGYDLSF.L	α-1	1189 - 1199

C-telopeptide	1434.69	1	P.GPPSGGYDLSFLPQ.P	α-1	1189 - 1202
C-telopeptide	1756.85	7	P.GPPSGGYDLSFLPQPPQ.E	α-1	1189 - 1205
C-telopeptide	1885.89	4	P.GPPSGGYDLSFLPQPPQE.K	α-1	1189 - 1206
C-telopeptide	2222.08	1	P.GPPSGGYDLSFLPQPPQEKAH.D	α-1	1189 - 1209
C-telopeptide	2337.12	1	P.GPPSGGYDLSFLPQPPQEKAHD.G	α-1	1189 - 1210
C-telopeptide	2394.14	2	P.GPPSGGYDLSFLPQPPQEKAHDG.G	α-1	1189 - 1211
C-telopeptide	2607.25	7	P.GPPSGGYDLSFLPQPPQEKAHDGGR.Y	α-1	1189 - 1213
C-telopeptide	2933.40	32	P.GPPSGGYDLSFLPQPPQEKAHDGGRYY.R	α-1	1189 - 1215
C-telopeptide	3160.52	5	P.GPPSGGYDLSFLPQPPQEKAHDGGRYYRA.D	α-1	1189 - 1217
C-telopeptide	3390.54	1	P.GPPSGGYDLSFLPQPPQEKAHDGGRYYRADD.A	α-1	1189 - 1219
C-telopeptide	2682.26	5	P.SGGYDLSFLPQPPQEKAHDGGRYY.R	α-1	1192 - 1215
C-telopeptide	1290.63	1	S.GGYDLSFLPQPP.Q	α-1	1193 - 1204
C-telopeptide	1418.69	1	S.GGYDLSFLPQPPQ.E	α-1	1193 - 1205
C-telopeptide	2595.20	3	S.GGYDLSFLPQPPQEKAHDGGRYY.R	α-1	1193 - 1215
C-telopeptide	2822.36	1	S.GGYDLSFLPQPPQEKAHDGGRYYRA.D	α-1	1193 - 1217
C-telopeptide	2002.98	1	S.FLPQPPQEKAHDGGRYY.R	α-1	1199 - 1215
triple-helical	1144.52	1	P.GKNGDDGEAGKP.G	α-1	226 - 237
triple-helical	1201.60	1	K.GHRGFSGLDGAK.G	α-1	265 - 276
triple-helical	1094.51	2	R.GFSGLDGAKGDA.G	α-1	268 - 279
triple-helical	851.42	1	R.GFSGLDGAK.G	α-1	268 - 276
triple-helical	1057.48	1	V.GPAGKDGEAGAQ.G	α-1	607 - 618
triple-helical	826.45	1	G.PQGIAGQR.G	α-1	950 - 957

triple-helical	1248.54	1	R.GDKGETGEQGDR.G	α-1	1093 - 1104
triple-helical	1546.74	1	R.GDKGETGEQGDRGIK.G	α-1	1093 - 1107
triple-helical	1896.92	2	R.GDKGETGEQGDRGIKGHR.G	α-1	1093 - 1110
triple-helical	2188.04	2	R.GDKGETGEQGDRGIKGHRGFS.G	α-1	1093 - 1113
triple-helical	2486.20	1	R.GDKGETGEQGDRGIKGHRGFSGLQ.G	α-1	1093 - 1116
triple-helical	801.38	1	R.GRTGDAGPA.G	α-1	1168 - 1176
C-telopeptide	1189.48	1	P.GPSGGGYEFGFD.G	α-2	1100 - 1111
C-telopeptide	1508.60	1	P.GPSGGGYEFGFDGDF.Y	α-2	1100 - 1114
C-telopeptide	1671.67	33	P.GPSGGGYEFGFDGDFY.R	α-2	1100 - 1115
C-telopeptide	1827.76	28	P.GPSGGGYEFGFDGDFYR.A	α-2	1100 - 1116
C-telopeptide	1898.80	53	P.GPSGGGYEFGFDGDFYRA.D	α-2	1100 - 1117
C-telopeptide	1517.58	1	P.SGGGYEFGFDGDFY.R	α-2	1102 - 1115
C-telopeptide	1673.68	2	P.SGGGYEFGFDGDFYR.A	α-2	1102 - 1116
C-telopeptide	1744.72	2	P.SGGGYEFGFDGDFYRA.D	α-2	1102 - 1117
C-telopeptide	1267.49	2	S.GGGYEFGFDGDF.Y	α-2	1103 - 1114
C-telopeptide	1430.55	26	S.GGGYEFGFDGDFY.R	α-2	1103 - 1115
C-telopeptide	1586.65	39	S.GGGYEFGFDGDFYR.A	α-2	1103 - 1116
C-telopeptide	1657.69	35	S.GGGYEFGFDGDFYRA.D	α-2	1103 - 1117
C-telopeptide	1373.53	2	G.GGYEFGFDGDFY.R	α-2	1104 - 1115
C-telopeptide	1529.64	3	G.GGYEFGFDGDFYR.A	α-2	1104 - 1116
C-telopeptide	1600.67	2	G.GGYEFGFDGDFYRA.D	α-2	1104 - 1117
C-telopeptide	1316.51	2	G.GYEFGFDGDFY.R	α-2	1105 - 1115

C-telopeptide	1472.61	1	G.GYEFGFDGDFYR.A	α-2	1105 - 1116
C-telopeptide	1543.65	3	G.GYEFGFDGDFYRA.D	α-2	1105 - 1117
C-telopeptide	1194.52	1	E.FGFDGDFYRA.D	α-2	1108 - 1117
C-telopeptide	919.39	1	G.FDGDFYR.A	α-2	1110 - 1116
C-telopeptide	990.43	2	G.FDGDFYRA.D	α-2	1110 - 1117
triple-helical	1108.58	1	K.GIRGHNGLDGL.K	α-2	176 - 186
triple-helical	1236.68	1	K.GIRGHNGLDGLK.G	α-2	176 - 187
triple-helical	1444.70	1	S.GEEGKRGSTGEIGPA.G	α-2	374 - 388
triple-helical	1188.63	2	P.GGKGEKGETGLR.G	α-2	650 - 661
triple-helical	846.37	1	A.GANGDRGEA.G	α-2	686 - 694
triple-helical	1071.48	1	A.GANGDRGEAGPA.G	α-2	686 - 697
triple-helical	891.40	1	P.GLAGHHGDQ.G	α-2	1031 - 1039

[a]Nano LC-MS was used to separate the peptides released from type I collagen fibers and to determine their molecular masses. The sequence of the released peptides was analyzed by Sequest HT search tool. Source data are provided as a Source Data file.
