# Supplementary material for: Structure of Vibrio collagenase VhaC provides insight into the mechanism of bacterial collagenolysis
Source: Nat Commun. 2022 Jan 28;13:566. doi: 10.1038/s41467-022-28264-1 (PMC8799719; doi:10.1038/s41467-022-28264-1)
Supplement: Supplementary file 2 — Description of Additional Supplementary Files [file 41467_2022_28264_MOESM2_ESM.pdf]

Description of Additional Supplementary Files

File Name: Supplementary Data 1

Description: Strains and plasmids used in this study.

File Name: Supplementary Data 2

Description: Primers used in this study.

File Name: Supplementary Movie 1

Description: The conformational opening and closing changes of unbound-CM in the 1000 ns molecular dynamics simulation (MDS).

File Name: Supplementary Movie 2

Description: The 1000 ns-MDS process of unbound-CM: THP binary complex. The triple helical collagen is close to the catalytic center of the peptidase domain with the conformation closing of CM.
