# Supplementary material for: Structure of Vibrio collagenase VhaC provides insight into the mechanism of bacterial collagenolysis
Source: Nat Commun. 2022 Jan 28;13:566. doi: 10.1038/s41467-022-28264-1 (PMC8799719; doi:10.1038/s41467-022-28264-1)
Supplement: Supplementary file 7 — Reporting Summary [file 41467_2022_28264_MOESM7_ESM.pdf]

nature portfolio

Corresponding author(s): Xiu-Lan Chen; Yu-Zhong Zhang

Last updated by author(s): Dec 15, 2021

Reporting Summary

Nature Portfolio wishes to improve the reproducibility of the work that we publish. This form provides structure for consistency and transparency in reporting. For further information on Nature Portfolio policies, see our Editorial Policies and the Editorial Policy Checklist.

Statistics

For all statistical analyses, confirm that the following items are present in the figure legend, table legend, main text, or Methods section.

n/a	Confirmed	
☐	☒	The exact sample size (n) for each experimental group/condition, given as a discrete number and unit of measurement
☐	☒	A statement on whether measurements were taken from distinct samples or whether the same sample was measured repeatedly
☐	☒	The statistical test(s) used AND whether they are one- or two-sided<br>*Only common tests should be described solely by name; describe more complex techniques in the Methods section.*
☒	☐	A description of all covariates tested
☒	☐	A description of any assumptions or corrections, such as tests of normality and adjustment for multiple comparisons
☐	☒	A full description of the statistical parameters including central tendency (e.g. means) or other basic estimates (e.g. regression coefficient) AND variation (e.g. standard deviation) or associated estimates of uncertainty (e.g. confidence intervals)
☐	☒	For null hypothesis testing, the test statistic (e.g. F, t, r) with confidence intervals, effect sizes, degrees of freedom and P value noted<br>*Give P values as exact values whenever suitable.*
☒	☐	For Bayesian analysis, information on the choice of priors and Markov chain Monte Carlo settings
☒	☐	For hierarchical and complex designs, identification of the appropriate level for tests and full reporting of outcomes
☒	☐	Estimates of effect sizes (e.g. Cohen's d, Pearson's r), indicating how they were calculated

Our web collection on statistics for biologists contains articles on many of the points above.

Software and code

Policy information about availability of computer code

Data collection	The x-ray diffraction data of the crystals were processed using HKL3000 v. 721.3.<br>The molecular dynamics simulation (MDS) was performed using software package GROMACS 2019.<br>The circular dichroism spectra were monitoried using JASCO Spectra Manager v. 2.15.01.<br>The isothermal titration calorimetry (ITC) measurement was performed using MicroCal PEAQ-ITC analysis software v.1.1.0.1262.<br>AFM imaging data were collected using Nanoscope v. 8.10.<br>All small angle x-ray scattering (SAXS) data processing were accomplished using BioXTAS RAW v.1.6.0.
Data analysis	The sedimentation coefficients and molecular weight were calculated by SEDFIT V14.4f.<br>The data of nano LC-MS were analyzed using Proteome Discoverer software 1.4.<br>The crystal structure was analyzed using the Phenix v. 1.19.2-4158, Coot v.0.8.9.2, and PyMol v.2.5.2.<br>The isothermal titration calorimetry (ITC) data were analyzed using Microcal PEAQ-ITC analysis software v. 1.1.0.1262.<br>Software for initial data processing was Microsoft Excel 2019, and subsequent analyses were carried out using OriginPro 8.5 v.b161 and OriginPro 2018C v. b9.5.1. 195.<br>The small angle x-ray scattering (SAXS) data were analyzed using ATSAS v. 3.0.3 software package.<br>AFM data analysis were performed with NanoscopeAnalysis v. 1.40.<br>The circular dichroism spectra were monitoried using JASCO Spectra Manager v. 2.15.01.

For manuscripts utilizing custom algorithms or software that are central to the research but not yet described in published literature, software must be made available to editors and reviewers. We strongly encourage code deposition in a community repository (e.g. GitHub). See the Nature Portfolio guidelines for submitting code & software for further information.

Data

Policy information about availability of data

All manuscripts must include a data availability statement. This statement should provide the following information, where applicable:

- Accession codes, unique identifiers, or web links for publicly available datasets
- A description of any restrictions on data availability
- For clinical datasets or third party data, please ensure that the statement adheres to our policy

All data supporting the findings of this study are available within the paper (and its Supplementary Information files). The structure data of CM have been deposited in the Protein Data Bank (PDB) database under accession code 7ESI [https://www.rcsb.org/structure/7ESI]. The SAXS data and models have been deposited in the Small Angle Scattering Biological Data Bank (SASBDB) under accession code SASDMU3 [https://www.sasbdb.org/data/SASDMU3]. Other structure data used in this study are available in the Protein Data Bank (PDB) database under accession code 2Y50 [https://www.rcsb.org/structure/2Y50], 2Y6I [https://www.rcsb.org/structure/2Y6I], 1K6F [https://www.rcsb.org/structure/1K6F], 4TN9 [https://www.rcsb.org/structure/4TN9] and 2LUW [https://www.rcsb.org/structure/2LUW]. The sequence data of VhaC and ColG used in this study are available in the GenBank database under accession codes WP_047516938.1 [https://www.ncbi.nlm.nih.gov/protein/WP_047516938.1/] and D87215.1 [https://www.ncbi.nlm.nih.gov/nuccore/D87215.1], respectively. A reporting summary for this article is available as a Supplementary Information file. Source data are provided with this paper.

Field-specific reporting

Please select the one below that is the best fit for your research. If you are not sure, read the appropriate sections before making your selection.

☒ Life sciences ☐ Behavioural & social sciences ☐ Ecological, evolutionary & environmental sciences

For a reference copy of the document with all sections, see nature.com/documents/nr-reporting-summary-flat.pdf

Life sciences study design

All studies must disclose on these points even when the disclosure is negative.

Sample size	No sample size calculations were performed for this study. Sample sizes are indicated for each experiment and were chosen based on similar studies. Where statistical tests were applied, we selected n=3 as the minimum sample size.
Data exclusions	No data was excluded from the analysis.
Replication	All experiments were repeated on at least 3 separate samples using the reported methods. All attempts at replication were successful.
Randomization	The designed experiments in this study didn't include random experiments, and the experimental results were not randomized.
Blinding	Blinding is not relevant to this study: only one variable is tested in each experiment

Reporting for specific materials, systems and methods

We require information from authors about some types of materials, experimental systems and methods used in many studies. Here, indicate whether each material, system or method listed is relevant to your study. If you are not sure if a list item applies to your research, read the appropriate section before selecting a response.

Materials & experimental systems

n/a	Involved in the study	
☒	☐	Antibodies
☒	☐	Eukaryotic cell lines
☒	☐	Palaeontology and archaeology
☒	☐	Animals and other organisms
☒	☐	Human research participants
☒	☐	Clinical data
☒	☐	Dual use research of concern

Methods

n/a	Involved in the study	
☒	☐	ChIP-seq
☒	☐	Flow cytometry
☒	☐	MRI-based neuroimaging
